# Cystine-Dependent Redox Metabolism Determines Photodynamic Therapy Response in Head and Neck Squamous Cell Carcinoma

**DOI:** 10.1021/acsnano.6c10240

**Published:** 2026-09-07

**Authors:** Amir Roshanzadeh, Hyllana C. D. Medeiros, Tessa Jordan, Carson Malhado, Katherine Espinoza Morelos, Mohamed Khalil, Jeyashri Muthukumar, Laura Favazza, Steven S. Chang, James M. Hamby, Laura C. Cesa, Dohun Pyeon, Vincent Lavallo, Richard R. Lunt, Sophia Y. Lunt

**Affiliations:** † Department of Biochemistry and Molecular Biology, 3078 Michigan State University, East Lansing, Michigan 48824, United States; ‡ Department of Chemical Engineering and Materials Science, Michigan State University, East Lansing, Michigan 48824, United States; § Department of Chemistry, 8790 University of California Riverside, Riverside, California 92521, United States; ∥ Department of Microbiology, Genetics, and Immunology, Michigan State University, East Lansing, Michigan 48824, United States; ⊥ Department of Molecular Biology, National Research Center, Giza 12622, Egypt; # Department of Pathology and Laboratory Medicine, Henry Ford Health, Detroit, Michigan 48202, United States; ∇ Department of Otolaryngology−Head and Neck Surgery, College of Human Medicine, Michigan State University, East Lansing, Michigan 48824, United States; ○ Department of Otolaryngology−Head and Neck Surgery, Henry Ford Health, Detroit, Michigan 48202, United States; ◆ Entrepreneur-in-Residence (EIR) Spartan Innovations, Michigan State University, East Lansing, Michigan 48824, United States; ¶ Department of Physics and Astronomy, Michigan State University, East Lansing, Michigan 48824, United States

**Keywords:** head and neck cancer, human papillomavirus, photodynamic therapy, near-infrared photosensitizers, oxidative stress, cancer therapeutics

## Abstract

Head and neck squamous cell carcinoma (HNSCC) remains difficult to treat without substantial functional and cosmetic morbidity, as current management relies largely on surgery, radiation, and chemotherapy. However, these approaches often fail to achieve effective tumor control in therapy-resistant HNSCC. Photodynamic therapy (PDT) has strong potential for HNSCC treatment because it enables spatially restricted tumor ablation while limiting damage to surrounding healthy tissue. Here, we apply a near-infrared (NIR) cyanine–carborate photosensitizer (PS) platform to HNSCC to identify a redox-based mechanism that limits PDT response in resistant cells and evaluate a strategy for sensitizing resistant cells. Using complementary human papillomavirus (HPV)-positive and -negative HNSCC models, we show that PDT induces acute oxidative stress and lipid peroxidation in both sensitive and resistant cells. However, resistant cells counter this stress through an adaptive redox response marked by increased xCT (SLC7A11)-dependent cystine utilization. This response sustains glutathione-linked antioxidant buffering, limits lipid peroxidation, and promotes survival under PDT. Genetic or pharmacologic disruption of cystine uptake prevents this adaptive defense, restores oxidative vulnerability, and sensitizes PDT-resistant HNSCC cells to treatment. Consistent with these mechanistic findings, cyanine–carborate-induced PDT with xCT (SLC7A11) inhibition markedly suppressed tumor growth *in vivo*. Together, these findings identify xCT-dependent cystine metabolism as a targetable vulnerability for therapeutic sensitization of resistant HNSCC to PDT.

## Graphical abstract

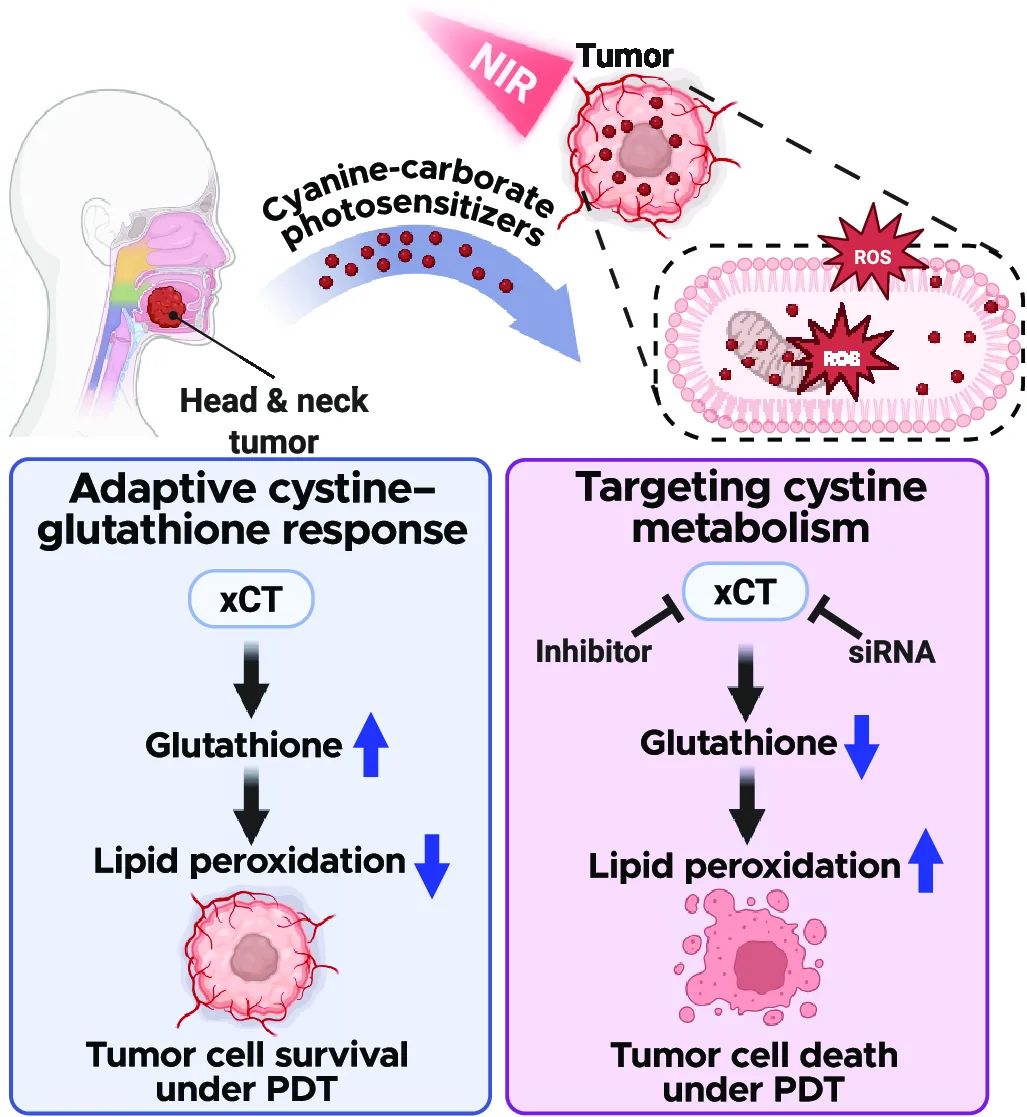

## Introduction

Head and neck squamous cell carcinoma (HNSCC) is a major global health burden,^1,2^ with approximately 947,000 new cases and 482,000 deaths reported worldwide in 2022. Survival remains poor for patients with advanced, recurrent, or treatment-refractory disease.^3,4^ Despite aggressive multimodal treatment strategies incorporating surgery, radiation, and chemotherapy, effective tumor control is frequently limited by therapeutic resistance and substantial treatment-associated functional and cosmetic morbidity^5^ that severely compromises quality of life, such as the ability to speak, taste, eat, smell, and breathe.^6^ Together, these challenges underscore a critical need for therapeutic approaches that achieve effective, localized tumor control while minimizing damage to the structurally complex and functionally essential tissues of the head and neck.

Photodynamic therapy (PDT) is a light-activated treatment that uses photosensitizers (PSs), light-responsive molecules that preferentially accumulate in tumor tissue, together with tissue oxygen and localized illumination to generate reactive oxygen species (ROS) within the illuminated field. This spatial control makes PDT a clinically attractive alternative or adjunct to conventional treatments for HNSCC, particularly for superficial or anatomically accessible lesions and for patients who are poor candidates for surgery or reirradiation.^7–9^ PDT with clinically approved PSs like porfimer sodium, temoporfin (mTHPC), and 5-aminolevulinic acid (5-ALA)–derived protoporphyrin IX has been used to treat superficial mucosal lesions and early neoplasia of the oral cavity and upper aerodigestive tract through PDT-mediated phototoxicity.^7–11^ While these agents have demonstrated feasibility and short-term tumor control in select clinical settings, their broader impact has been constrained by limited tumor selectivity, shallow light penetration, prolonged photosensitivity, and incomplete tumor response, and pronounced interpatient variability in the therapeutic response.^8,9,12^ Importantly, this variability likely reflects both the limitations of currently approved PSs and the biological complexity of HNSCC, where tumor-intrinsic redox programs can reduce oxidative injury and limit treatment response.^13^

Variable PDT response may also arise from the biological complexity of HNSCC. This disease is highly heterogeneous, with human papillomavirus (HPV) status representing one major determinant of tumor behavior and treatment response.^14–17^ HPV-positive (HPV^+^) tumors, most commonly arising in the oropharynx, often present in middle-aged patients at a relatively younger age than HPV-negative (HPV^–^) disease and show improved responses to chemoradiation.^15,16,18^ These differences have been attributed in part to viral oncogene–driven rewiring of tumor-suppressor signaling, immune dysregulation, and broader metabolic and redox remodeling, including altered oxidative metabolism and glutathione (GSH) homeostasis.^19,20^ Clinically, resistant HPV^–^ HNSCC remains difficult to treat. Immune checkpoint inhibitor–based systemic therapies, including pembrolizumab-based regimens, have improved treatment options for metastatic disease.^21^ However, 5-year overall survival remains only ∼15–16%, and targeted options after progression remain limited. The activity of tipifarnib in *HRAS* mutant HNSCC shows that defined molecular dependencies can be exploited therapeutically, but such approaches apply only to selected patient subset.^22^ Despite growing interest in molecularly defined vulnerabilities, the metabolic mechanisms that determine whether PDT-induced oxidative stress culminates in tumor cell death or adaptive survival remain poorly defined.

Oxidative stress is a central mediator of PDT-induced phototoxicity, raising the possibility that differences in redox regulation critically shape therapeutic outcomes in HNSCC.^23^ Cancer cells possess highly plastic antioxidant networks that buffer ROS and limit oxidative damage to lipids, proteins, and nucleic acids, enabling survival under otherwise lethal oxidative stress through rapid stress-adaptive transcriptional programs.^24,25^ Previously, we developed a chemically tunable near-infrared (NIR) cyanine–carborate photosensitizer (PS) platform with enhanced photostability, tumor targeting, and photodynamic efficiency.^26–28^ Here, we use this next-generation platform to compare how complementary HNSCC models with differential PDT response handle photodynamic oxidative stress. We identify a redox-metabolic adaptation mechanism centered on cystine utilization and GSH buffering that determines whether PDT-induced oxidative stress is translated into cell death or adaptive survival.^29^ This program sustains glutathione-dependent redox homeostasis, limits lipid peroxidation, and promotes early survival following PDT, particularly in resistant HNSCC models. Disruption of cystine uptake collapses this protective state, establishing cystine-dependent GSH metabolism as a targetable metabolic vulnerability for improving PDT response in resistant HNSCC.^30^

## Results

### Cyanine–Carborate PDT Reveals Heterogeneous Sensitivity across HNSCC Models

To compare PDT response in treatment-sensitive versus treatment-resistant HNSCC models, we employed an established NIR cyanine–carborate photosensitizer platform in which the counterion identity modulates photodynamic activity. Two representative cyanine–carborate salts incorporating CB_11_H_6_Cl_6_^–^ or CB_9_HCl_9_^–^ counterions that exhibit robust light-dependent activity and minimal dark cytotoxicity were used to systematically assess PDT responses across a panel of HPV^+^ (SCC2, SCC90, SCC152) and HPV^–^ (SCC1, SCC4, SCC19) HNSCC cell lines (Figures 1A and S1).^26^ Here, phototoxicity refers to toxicity following NIR-mediated PS activation, whereas cytotoxicity refers to light-independent toxicity under dark conditions.

**Figure 1:**
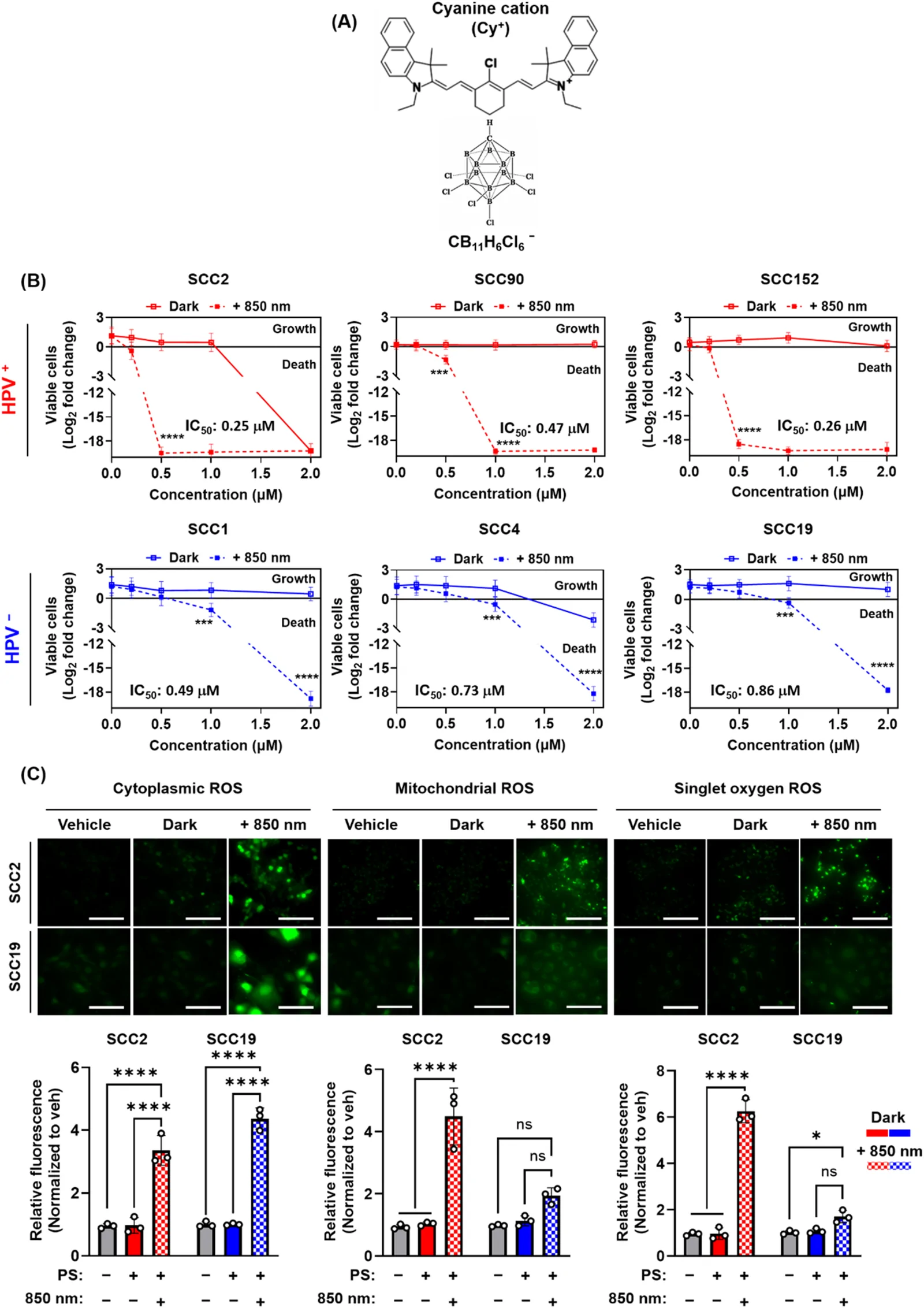
Photodynamic activity and oxidative stress responses of cyanine-carborate PSs in HNSCC. (A) Chemical structures of the cyanine cation paired with CB_11_H_6_Cl_6_^–^ carborate counterion used as near-infrared (NIR) PSs. (B) Concentration–response analysis of cell viability following cyanine–carborate treatment under dark conditions or NIR (850 nm) irradiation in HPV^+^ (SCC2, SCC90, SCC152) and HPV^–^ (SCC1, SCC4, SCC19) HNSCC cell lines. IC_50_ values shown represent NIR–dependent phototoxicity; corresponding dark cytotoxic IC_50_ values are provided in Table S2. (C) Representative fluorescence images and quantitative analysis of cytoplasmic ROS, mitochondrial ROS, and singlet oxygen production following PDT in representative HPV^+^ (SCC2) and HPV^–^ (SCC19) cell lines. Scale bar: 70 μm. Data are presented as the mean ± SD (*n* = 3; *P < 0.05, ****P < 0.0001; ns, not significant).

Following NIR irradiation at 850 nm, concentration–response analysis revealed heterogeneous PDT sensitivity across the HNSCC panel, with several HPV^–^ models displaying greater resistance than HPV^+^ counterparts (Figure 1B). In HPV^+^ cell lines, cyanine–carborate treatment under light resulted in a steep and concentration-dependent reduction in viability. At lower PS concentrations (i.e., 0.1–0.5 μM), HPV^+^ cells exhibited a marked loss of viability, decreasing from near-baseline levels in dark conditions to approximately 15–30% following irradiation, corresponding to a 3–6-fold reduction in viable cells (Figure 1B). Increasing the PS concentration beyond this range produced only modest additional loss of viability, indicating that maximal phototoxic responses were achieved at relatively low concentrations. In contrast, several HPV^–^ HNSCC cell lines showed attenuated responses to PDT under identical conditions. Across the same concentration range (0.1–0.5 μM), HPV^–^ cells showed a more limited reduction in viability following NIR irradiation, typically retaining 60–80% viability relative to dark-treated controls (Figure 1B). This resistance phenotype was most evident in SCC19 cells, which retained substantial viability across the tested concentration range and exhibited the highest phototoxic half-maximal inhibitory concentration (IC_50_) among all tested HNSCC models (approximately 0.86 μM; Table S2). In contrast, HPV^+^ cell lines generally demonstrated greater PDT sensitivity, with SCC2 showing the lowest IC_50_ (approximately 0.25 μM), followed by SCC152 (approximately 0.26 μM) and SCC90 (approximately 0.47 μM). The HPV^–^ SCC1 and SCC4 cell lines showed intermediate responses, with IC_50_ values of approximately 0.49 and 0.73 μM, respectively. Across all cell lines, dark-treated controls maintained near-baseline viability, indicating minimal light-independent cytotoxicity of the PSs. Based on the complete NIR phototoxic concentration–response profiles and corresponding IC_50_ values, SCC2 and SCC19 were selected as representative PDT-sensitive and PDT-resistant models, respectively, for subsequent mechanistic studies (Figure 1B and Table S2). These terms represent relative classifications within the tested HNSCC panel under the matched experimental conditions and were not based on viability at a single PS concentration, a predefined universal IC_50_ threshold, or HPV status alone. A second cyanine–carborate pairing, CyCB_9_HCl_9_, with similar energy level alignment reproduced the same overall response pattern across the HPV^+^ and HPV^–^ panel, indicating that the observed sensitivity hierarchy was not limited to a single PS formulation (Figure S1). We then confirmed PS uptake and subcellular distribution in representative sensitive and resistant models (Figure S2A). In both models, the PSs localized predominantly to mitochondria, with additional detectable signal in the endoplasmic reticulum, Golgi apparatus, and lysosomes (Figure S2B).

Given that ROS generation is a central mediator of PDT-induced phototoxicity,^33,34^ we next assessed whether differences in oxidative stress induction accompanied the divergent viability responses. Using live-cell fluorescence imaging, cytoplasmic ROS, mitochondrial ROS, and singlet oxygen production were quantified following cyanine–carborate PDT in representative HPV^+^ (SCC2) and HPV^–^ (SCC19) cell lines (Figure 1C). In SCC2 cells, NIR irradiation in the presence of cyanine–carborate PSs resulted in a robust oxidative stress across all measured compartments. Cytoplasmic ROS levels increased by approximately 4–6-fold relative to dark-treated controls, mitochondrial ROS increased by ∼3–5-fold, and singlet oxygen production rose to ∼ 5-fold above baseline (Figure 1C). In contrast, SCC19 cells exhibited a selectively attenuated oxidative stress response under identical treatment conditions. Following PDT, cytoplasmic ROS levels in SCC19 increased by approximately 4.2-fold relative to dark-treated controls, whereas no significant induction of mitochondrial ROS or singlet oxygen was detected. These compartment-restricted ROS responses were evident at early postirradiation time points and occurred prior to measurable loss of cell viability, indicating that PDT-sensitive SCC2 and PDT-resistant SCC19 cells differ early in compartment-specific oxidative stress handling during PDT. Together, these quantitative differences indicate that PDT responsiveness is associated with differences in the magnitude and subcellular distribution of ROS after irradiation. Sensitive SCC2 cells exhibited broad cytoplasmic, mitochondrial, and singlet oxygen responses, whereas resistant SCC19 cells showed a more restricted ROS profile and survived under photodynamic conditions that were phototoxic to SCC2 cells.

### Differential Stress-Response Signaling Accompanies PDT Sensitivity and Resistance in HNSCC

Considering the divergent oxidative stress and survival responses following PDT, we examined whether canonical cellular stress-response pathways were differentially engaged in SCC2 and SCC19 cells, which represent PDT-sensitive HPV^+^ and PDT-resistant HPV^–^ HNSCC models, respectively. Nuclear factor erythroid 2-related factor 2 (Nrf2), encoded by *NFE2L2*, and activating transcription factor 4 (ATF4) are central regulators of oxidative stress responses and amino acid metabolism.^35,36^ Nrf2 activity is regulated in part by Kelch-like ECH-associated protein 1 (Keap1), a redox-sensitive adaptor that restrains Nrf2 signaling under basal conditions. Both pathways are implicated in oxidative stress adaptation,^36^ prompting evaluation of early and intermediate gene-expression and protein-level changes associated with Nrf2, ATF4, and downstream redox-adaptive targets following PDT exposure in SCC2 and SCC19 cells.

To define the temporal dynamics of stress-response activation, transcriptional responses were assessed at early (2 h) and intermediate (6 h) time points following PDT in SCC2 and SCC19 cells (Figure 2A,B). *HMOX1* encodes heme oxygenase 1 (HMOX-1), a canonical oxidative-stress response protein involved in redox adaptation.^37^ At 2 h post-PDT, SCC2 exhibited coordinated induction of Nrf2-associated stress-response transcripts, with *NFE2L2* increasing to approximately 2.4–2.6-fold and *HMOX-1* increasing to approximately 1.8–2.0-fold relative to dark-treated controls (Figure 2A). In contrast, SCC19 showed no significant induction of these transcripts at this early time point. *ATF4* mRNA increased modestly in SCC2 (∼1.4–1.6-fold) but remained unchanged in SCC19, further distinguishing early transcriptional responses between sensitive and resistant models. By 6 h post-PDT (Figure 2B), *HMOX1* expression remained elevated in SCC2, increasing approximately 2.4–2.7-fold. In SCC19, Nrf2-pathway engagement remained attenuated despite a significant reduction in *KEAP1* expression to approximately 0.5–0.6-fold under irradiation. *ATF4* transcriptional responses were temporally restricted, with decreased expression under irradiation at 6 h in both models, indicating that early transcriptional activation does not persist at later time points.

**Figure 2:**
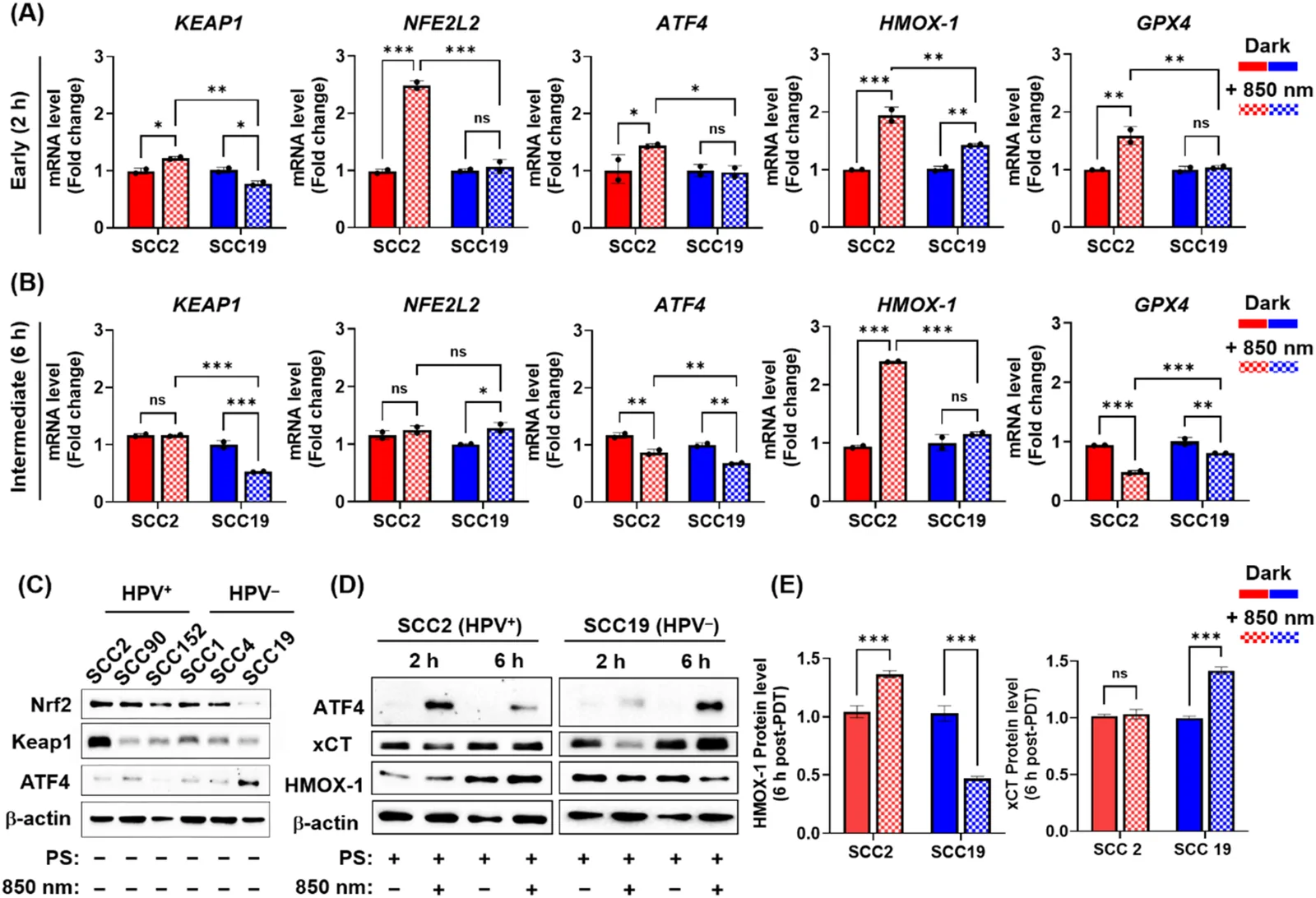
Dynamic stress-response signaling after photodynamic therapy in HNSCC. (A, B) qPCR analysis of stress-response gene expression at early (2 h; A) and intermediate (6 h; B) time points following PDT in representative HPV^+^ (SCC2) and HPV^–^ (SCC19) HNSCC cell lines. Transcript levels of *KEAP1*, *NFE2L2*, *ATF4*, *HMOX-1*, and *GPX4* are shown under dark conditions or following NIR (850 nm) irradiation. Data are presented as mean ± SD (*n* = 3; *P < 0.05, **P < 0.01, ***P < 0.001; ns, not significant). (C) Basal protein expression of Nrf2, Keap1, and ATF4 across a panel of HPV^+^ (SCC2, SCC90, SCC152) and HPV^–^ (SCC1, SCC4, SCC19) HNSCC cell lines. β-actin is used as a loading control. (D) Immunoblot analysis of ATF4, xCT, and HMOX-1 expression in SCC2 (HPV^+^) and SCC19 (HPV^–^) cells at 2 and 6 h following PDT. (E) Quantification of HMOX-1 and xCT protein levels at 6 h post-PDT, normalized to β-actin. Data are presented as mean ± SD (***P < 0.001; ns, not significant).

Since nuclear localization more directly reflects Nrf2 transcriptional activity than total protein abundance, nuclear Nrf2 was examined in SCC2 cells at 2 and 6 h after PDT. Although *NFE2L2* and *HMOX1* transcript levels increased at the early 2-h time point, nuclear-fraction immunoblotting showed no corresponding increase in nuclear Nrf2. Instead, nuclear Nrf2 levels decreased following NIR irradiation at both 2 and 6 h, with a greater reduction at 6 h (Figure S3). Thus, the early Nrf2-associated transcriptional response in SCC2 cells was not accompanied by sustained nuclear Nrf2 accumulation. Protein-level analyses further revealed distinct stress-response states in PDT-sensitive and PDT-resistant HNSCC cells. xCT, encoded by *SLC7A11*, imports cystine to support intracellular cysteine availability and GSH synthesis. Its induction in resistant cells could therefore contribute to the maintenance of antioxidant capacity following PDT. Across the broader HNSCC panel, including HPV^+^ SCC2, SCC90, and SCC152 cells and HPV^–^ SCC1, SCC4, and SCC19 cells, basal total Nrf2 and ATF4 abundance varied substantially (Figure 2C). SCC2, the most PDT-sensitive cell line, exhibited higher basal total Nrf2 abundance, whereas SCC19, the most PDT-resistant cell line, showed elevated basal ATF4 abundance, indicating distinct baseline stress-response states. Consistent with the transcriptional data, HMOX-1 protein increased by approximately 1.3-fold in SCC2 but decreased in SCC19 following irradiation (Figure 2D,E). In contrast, xCT protein remained unchanged in SCC2 but was selectively induced in SCC19, increasing to approximately 1.4–1.5-fold relative to dark-treated controls (Figure 2D,E). Collectively, these data show that SCC2 and SCC19 engage distinct stress-response pathways after PDT. SCC2 exhibited transient Nrf2-associated signaling, whereas SCC19 selectively induced xCT, suggesting a potential shift toward cystine-linked redox adaptation during PDT.

### xCT-Dependent Cystine Metabolism Suppresses Lipid Peroxidation and Enables PDT Resistance

Since xCT was selectively induced in resistant SCC19 cells after PDT, we next asked whether xCT-dependent cystine transport supports redox adaptation and limits lipid peroxidation during PDT in HPV^–^ HNSCC. xCT (*SLC7A11*) mediates cystine import, which sustains intracellular cysteine availability for GSH synthesis (Figure 3A).^38^ This pathway could therefore provide resistant cells with a mechanism to preserve antioxidant capacity and limit lipid ROS during PDT. Pharmacologic inhibition of xCT using the selective inhibitor imidazole ketone erastin (IKE)^30^ revealed a marked dependency on this pathway for SCC19 cell survival under PDT (Figure 3B). Whereas IKE alone under dark conditions produced a gradual, concentration-dependent decline in viability (∼60–70% survival at 1.6 μM), combining IKE with NIR irradiation resulted in a steep loss of viability, reducing survival to ∼10% at the same concentration. Consistent with this interpretation, extracellular cystine withdrawal through nutrient deprivation reduced viability under basal conditions without pharmacologic xCT inhibition (Figure S4), but the magnitude of this effect was substantially smaller than the loss observed when cystine limitation was combined with PDT. To determine whether this xCT-associated vulnerability extended beyond SCC19, we examined additional HNSCC models. PDT increased xCT expression in HPV^–^ resistant SCC4 cells, whereas xCT induction was limited in HPV^+^ sensitive SCC152 cells. Moreover, IKE concentration-dependently enhanced PDT sensitivity in SCC4 cells (Figure S5). These results support xCT-linked cystine metabolism as a resistance-associated vulnerability in HPV^–^ HNSCC models.

**Figure 3:**
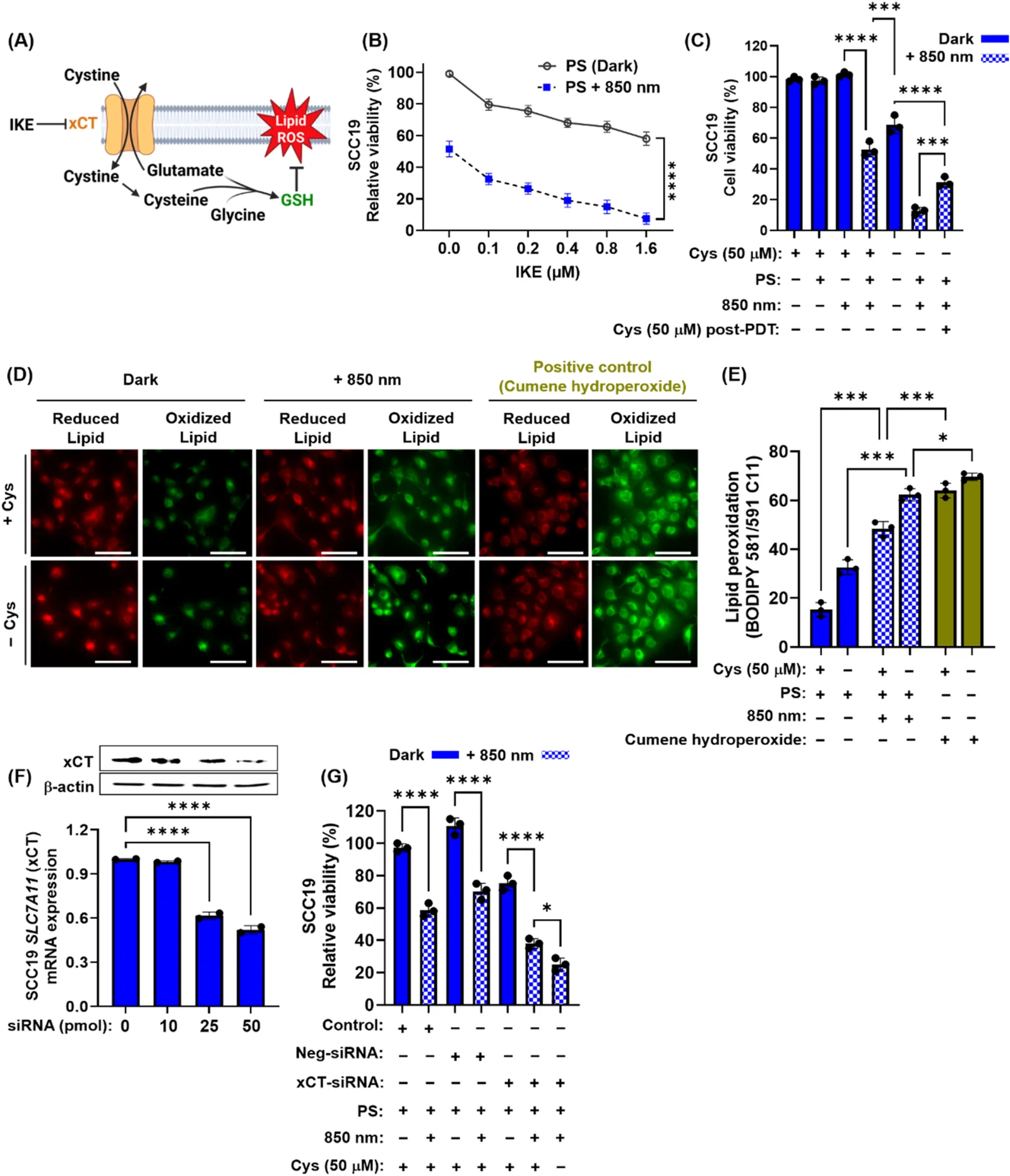
xCT-linked cystine availability and its impact on lipid peroxidation during PDT in resistant HNSCC. (A) Schematic illustrating xCT-mediated cystine uptake and its contribution to intracellular cysteine and GSH synthesis. (B) Viability of SCC19 cells treated with increasing concentrations of the xCT inhibitor IKE under dark conditions or following NIR (850 nm) irradiation. (C) Viability of SCC19 cells cultured under physiological cystine conditions (50 μM) or in cystine-free media over time in the absence of irradiation. (D) Representative fluorescence images of reduced and oxidized lipids in SCC19 cells cultured with or without cystine under dark conditions or following PDT. Cumene hydroperoxide-treated cells are shown as a positive control for lipid peroxidation. Scale bar: 70 μm. (E) Quantification of lipid peroxidation measured by BODIPY 581/591 C11 oxidation under the indicated conditions in SCC19 cells. The two green bars represent cumene hydroperoxide-treated cells used as a positive control for maximal lipid peroxidation with or without cystine. (F) Immunoblot and qPCR validation of different concentrations of siRNA-mediated knockdown of (xCT) *SLC7A11* in SCC19 cells. (G) Viability of SCC19 cells following PDT with or without xCT knockdown and cystine supplementation after 24 h.

We next examined intrinsic dependence on extracellular cystine (Figure 3C). Under a physiologically relevant cystine concentration (50 μM),^39^ SCC19 cells maintained ∼100% viability, whereas cystine withdrawal reduced survival to ∼60% at 24 h, indicating basal reliance on cystine-supported redox homeostasis. We then asked whether this dependence became more pronounced during PDT (Figure 3D). Following NIR irradiation, cystine-replete cells retained ∼55% viability, whereas cystine deprivation reduced survival further to ∼15%, corresponding to an overall ∼85% loss relative to baseline. Notably, resupplementation of cystine after PDT partially restored viability by ∼40%, demonstrating that extracellular cystine availability actively supports recovery from photodynamic injury. Consistent with these survival outcomes, lipid peroxidation analysis showed moderate oxidation (∼40–50% oxidized lipid fraction) in cystine-replete cells following PDT, whereas cystine deprivation increased oxidation to ∼60–70% (Figure 3D,E). Together, these findings establish cystine availability as a determinant of lipid redox control during PDT.

Genetic disruption of xCT-driven cystine uptake recapitulated the effects observed with pharmacologic inhibition. Small interfering RNA (siRNA)-mediated depletion of *SLC7A11* significantly reduced xCT mRNA and protein expression at 50 pmol (Figure 3F). Functionally, xCT knockdown alone reduced baseline viability by ∼35% (Figure 3G). Upon NIR activation, xCT depletion markedly enhanced sensitivity to PDT, decreasing cellular viability by ∼65% relative to control-transfected cells (Figure 3G). Combined xCT knockdown and cystine withdrawal further exacerbated cell death and reduced cell viability by ∼75%, underscoring the importance of cystine import in maintaining redox resilience during oxidative stress. Conversely, overexpression of xCT (*SLC7A11*) in SCC2 cells partially protected cells from PDT-induced loss of viability (Figure S6). This gain-of-function result complements the xCT knockdown data in SCC19 and indicates that increased xCT expression can partially enhance resistance to PDT. To further define the cell death pathways engaged when xCT-dependent cystine metabolism was disrupted, we performed pathway-directed inhibitor studies following PDT (Figure S7). Inhibition of necroptosis or autophagy did not significantly restore survival. In contrast, the pan-caspase inhibitor z-VAD-fmk (zVAD)^40^ and the lipid peroxidation inhibitor Ferrostatin-1 ^41^ each significantly rescued viability. Their combination produced the greatest protective effect, restoring SCC19 viability to approximately 95–100%, near dark-control levels (Figure S7). These results suggest that both apoptosis-associated and lipid peroxidation-associated cell death pathways contribute to PDT sensitivity when xCT-dependent cystine metabolism is disrupted. Collectively, these findings demonstrate that xCT-linked cystine availability restrains lipid peroxidation and sustains survival during PDT, and that collapse of this adaptive redox circuit renders PDT-resistant HNSCC cells susceptible to apoptosis- and lipid peroxidation–associated cell death under PDT.

### Altered Cysteine–Glutathione Metabolism Drives Differential PDT Sensitivity in HNSCC

To test whether PDT sensitivity was associated with distinct redox states, we quantified cysteine abundance in representative SCC2 and SCC19 HNSCC cells. In parallel, NADPH/NADP^+^, NADH/NAD^+^, and GSH/GSSG ratios were measured under dark conditions and following NIR irradiation, with or without pharmacological xCT inhibition (Figures 4A,B and S8). In SCC2 cells, intracellular cysteine decreased to ∼0.6-fold of baseline after PDT and to ∼0.3-fold when IKE was combined with PDT (Figure 4A). This cysteine reduction was accompanied by redox-state changes consistent with reduced buffering capacity. After PDT, the NADPH/NADP^+^ ratio decreased from ∼1.0 to ∼0.4, and the GSH/GSSG ratio decreased from ∼1.0 to ∼0.45. When IKE was combined with PDT, the NADPH/NADP^+^ and GSH/GSSG ratios remained below baseline at ∼0.7 and ∼0.8–0.9, respectively. In contrast, the NADH/NAD^+^ ratio changed only modestly after PDT alone, remaining ∼0.8–0.9, but decreased substantially to ∼0.4–0.5 when IKE was combined with PDT. Together, these data indicate that SCC2 cells undergo cysteine depletion together with loss of NADPH- and glutathione-linked reducing capacity during PDT.

**Figure 4:**
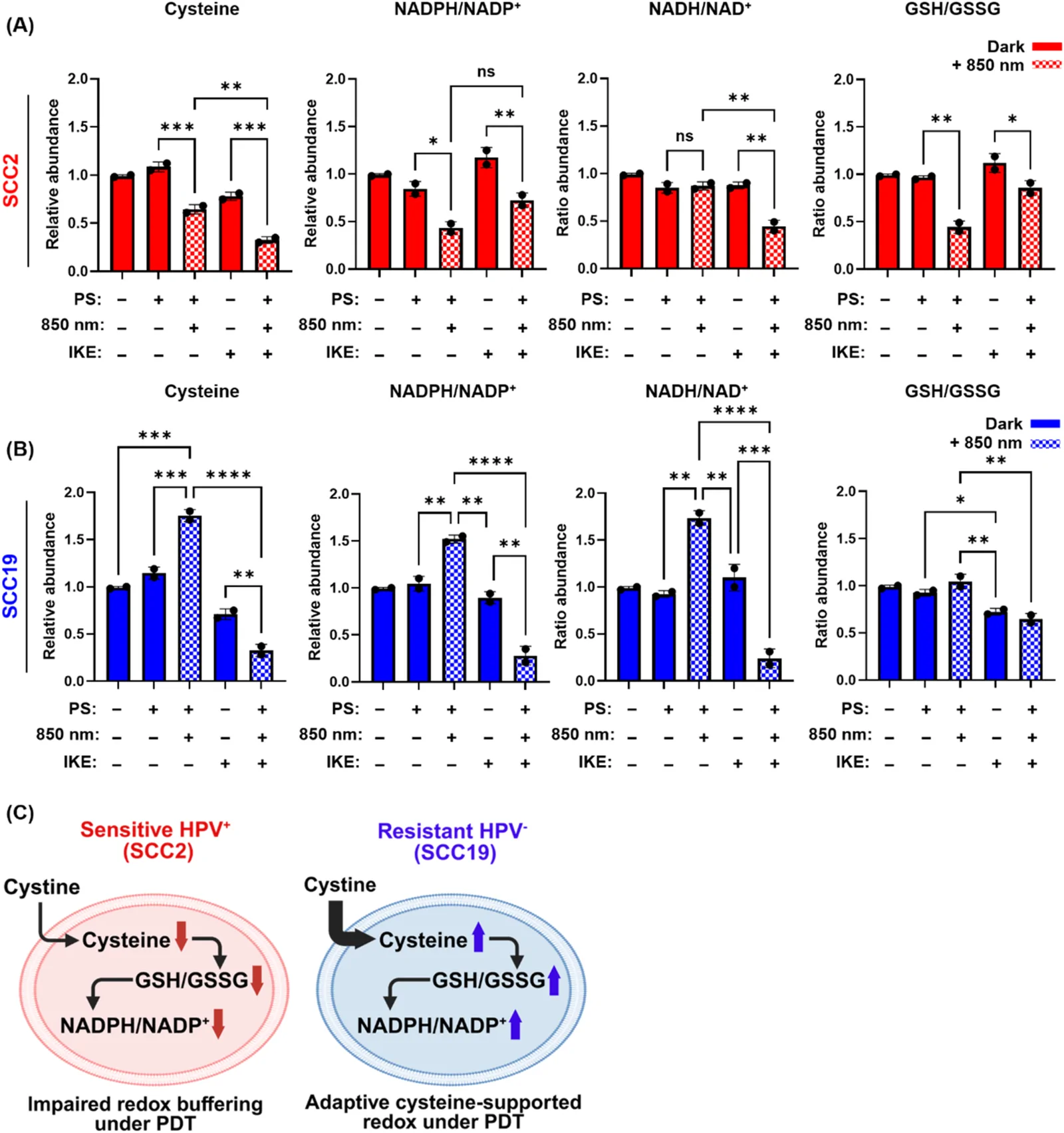
Redox metabolic profiling reveals distinct cysteine–glutathione buffering states in PDT-sensitive and PDT-resistant HNSCC. (A, B) Targeted LC–MS/MS metabolite profiling of intracellular cysteine, NADPH/NADP^+^, NADH/NAD^+^, and GSH/GSSG ratios in PDT-sensitive SCC2 cells (A) and PDT-resistant SCC19 cells (B) under dark conditions or following NIR irradiation, with or without xCT inhibition by imidazole ketone erastin (IKE). Metabolite signals were normalized to internal standards and subsequently to total protein abundance. Redox ratios are shown relative to dark-treated controls. Data are presented as mean ± SD (*n* = 3; *P < 0.05, **P < 0.01, ***P < 0.001; ns, not significant). (C) Working model summarizing the differential redox metabolic responses to PDT in sensitive SCC2 and resistant SCC19 cells.

In SCC19 cells, PDT triggered a distinct adaptive redox response, increasing intracellular cysteine from ∼1.0 to ∼1.7–1.8-fold relative to dark-treated controls (Figure 4B). IKE alone reduced cysteine to ∼0.7-fold, and combining IKE with PDT suppressed cysteine further to ∼0.3-fold, indicating that cysteine accumulation during photodynamic stress depends, at least in part, on xCT activity. In parallel, the NADPH/NADP^+^ ratio increased from ∼1.0 to ∼1.5 after PDT, suggesting increased NADPH-linked reducing capacity. This response was lost when xCT was inhibited, with the NADPH/NADP^+^ ratio decreasing to ∼ 0.3 under combined IKE and PDT conditions. The NADH/NAD^+^ ratio followed a similar pattern, increasing to ∼1.7 after PDT but decreasing to ∼0.2–0.3 when IKE was combined with PDT. The GSH/GSSG ratio was largely maintained or modestly increased after PDT but decreased to ∼0.6–0.7 when IKE was combined with PDT. Collectively, these findings reveal a metabolic dichotomy in PDT response (Figure 4C): sensitive SCC2 cells show cysteine depletion and loss of reduced redox-buffering capacity, whereas resistant SCC19 cells increase cysteine abundance and preserve NADPH, NADH, and glutathione-linked reducing capacity after PDT. xCT inhibition disrupts this adaptive state and converts SCC19 cells toward a redox-collapsed, PDT-sensitive phenotype.

### Cyanine–Carborate PDT Suppresses Tumor Growth in Subcutaneous Tumor Models

To evaluate the therapeutic efficacy and safety of cyanine–carborate PDT *in vivo*, we next examined PDT responses in subcutaneous flank HPV^+^ and HPV^–^ HNSCC models. In both HPV^+^ mEERL and HPV^–^ MOE/shPtpn14, *in vivo* fluorescence imaging revealed preferential accumulation and retention of the PSs at tumor sites following systemic administration, with signal intensity persisting up to 48 h postinjection and minimal background signal in surrounding tissue (Figure 5A). Tumor-associated signal remained detectable at 72 h (Figure S9A), supporting sustained local retention over the treatment window, while body weight remained stable across groups throughout the study (Figure S9B). *Ex vivo* imaging at day 20 revealed residual photosensitizer-associated fluorescence in several tissues, with prominent renal signal and lower or variable fluorescence in the liver, spleen, heart, and excised skin/tumor-associated tissue (Figure S9C). Consistent with this localization, PDT produced pronounced tumor-growth suppression *in vivo*. In the HPV^+^ mEERL model, mice receiving PSs with NIR irradiation exhibited a marked attenuation of tumor progression, with tumor volumes remaining below ∼100–150 mm^3^ at study end point, compared with ∼600–750 mm^3^ in vehicle-treated or nonirradiated control groups (Figure 5B). Notably, neither PS alone nor NIR irradiation alone produced substantial growth inhibition, indicating that PS-mediated tumor control required both PS administration and NIR irradiation. In the HPV^–^ MOE/shPtpn14 model, PS-mediated PDT also reduced tumor growth (Figure 5C), although the effect was less pronounced than that observed in HPV^+^ mEERL tumors. End point MOE/shPtpn14 tumor volumes remained ∼250–350 mm^3^ after PS-mediated PDT (Figure 5C), compared with ∼600–700 mm^3^ in the vehicle + NIR and PS dark control groups. To determine whether xCT inhibition further enhanced this response, mice were treated with IKE alone or in combination with PS-mediated PDT. IKE alone partially reduced tumor growth, whereas IKE plus PS-mediated PDT produced the greatest reduction in tumor volume, limiting end point tumors to approximately 100–150 mm^3^ (Figure 5C).

**Figure 5:**
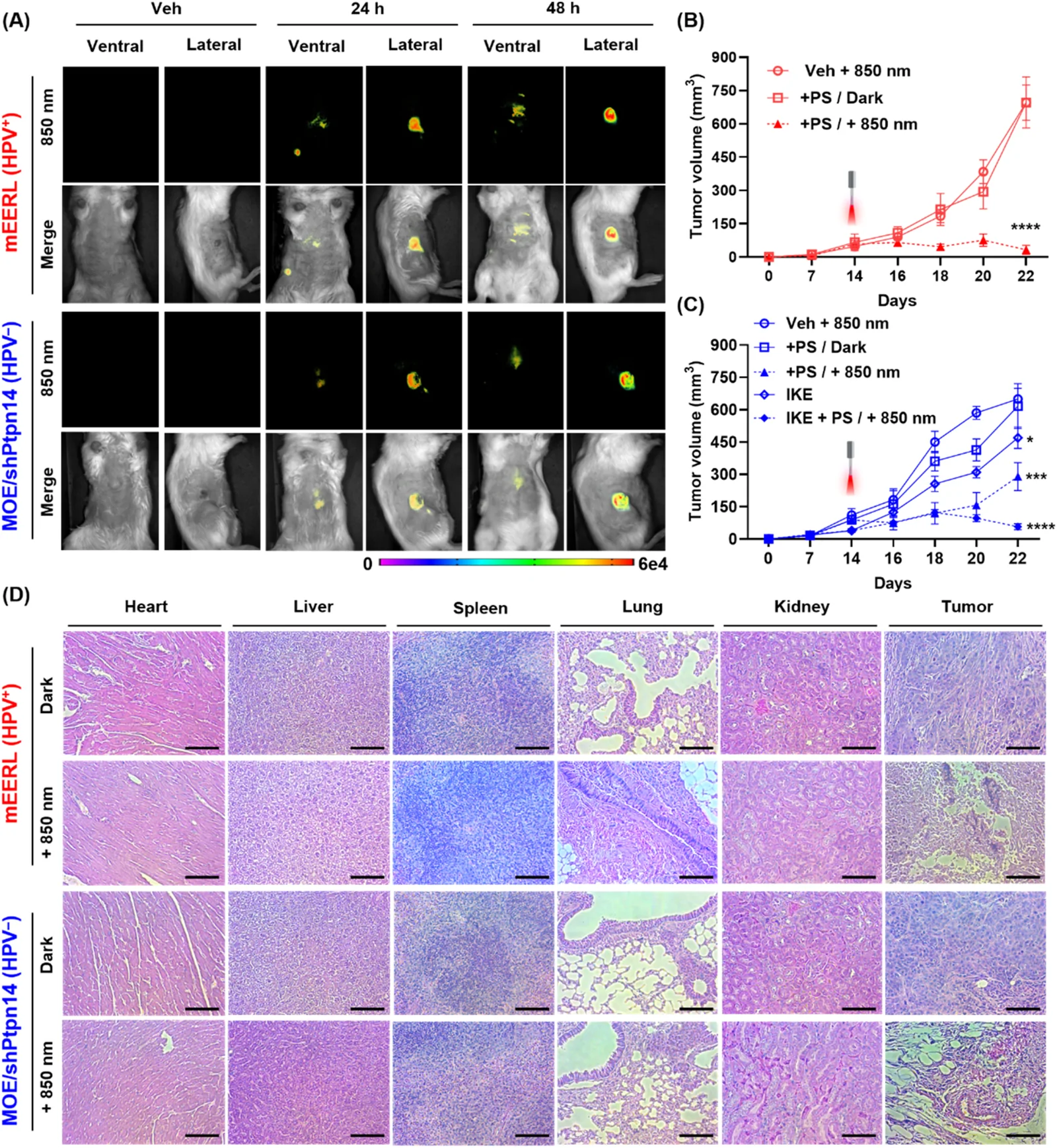
*In vivo* photodynamic therapy efficacy and tissue response in HPV^+^ and HPV^–^ HNSCC models. (A) Representative *in vivo* fluorescence and brightfield images showing biodistribution and tumor localization of the cyanine–carborate PS following intravenous administration in subcutaneous flank HPV^+^ (mEERL) and HPV^–^ (MOE/shPtpn14) tumor–bearing mice at indicated time points (vehicle, 24 and 48 h postinjection). Ventral views predominantly reflect hepatic uptake, whereas lateral views highlight PS accumulation at flank tumor sites. Merged images are shown under NIR (850 nm) excitation. (B) Tumor growth curves for mEERL (HPV^+^) tumors in mice treated with vehicle plus NIR irradiation (Veh + 850 nm), PS under dark conditions (PS/Dark), or PS followed by NIR irradiation (PS + 850 nm). (C) Tumor growth curves for MOE/shPtpn14 (HPV^–^) tumors in mice treated with vehicle plus NIR irradiation (Veh +850 nm), PS under dark conditions (PS/Dark), PS followed by NIR irradiation (PS + 850 nm), daily oral IKE (50 mg/kg), or IKE combined with PS-mediated PDT (IKE + PS + 850 nm). Tumor volumes were measured longitudinally and expressed as mean ± SD (*n* = 8; ****P < 0.0001) (D) Representative hematoxylin and eosin (H&E)–stained sections of heart, liver, spleen, lung, kidney, and tumor tissue collected from mEERL (HPV^+^) and MOE/shPtpn14 (HPV^–^) tumor–bearing mice under dark conditions or following PDT (+850 nm), illustrating tissue morphology and treatment-associated histopathology. Scale bar: 400 μm.

At the study end point, tissue-level responses and systemic tolerability were further examined. Immunohistochemical staining showed detectable xCT, Nrf2, and ATF4 expression within residual viable tumor regions following PDT, although extensive treatment-associated disruption of tumor architecture limited reliable quantitative comparison with dark controls (Figure S10). Independent histopathological evaluation showed no scored treatment-associated abnormalities in the heart, liver, spleen, lung, or kidney, with combined major-organ scores of 0.0 ± 0.0 in both dark-control and NIR-treated groups (Table S3). Occasional focal or mild chronic inflammatory findings were noted in individual sections but were not accompanied by positive histopathological injury scores. In contrast, NIR-treated tumors exhibited a histopathological score of 4.0 ± 0.0 compared with 0.0 ± 0.0 in dark-control tumors, consistent with pronounced localized tumor injury following PDT (Figure 5D and Table S3). Serum alanine aminotransferase (ALT) activity remained unchanged across treatment groups, and NIR irradiation did not increase aspartate aminotransferase (AST) activity in either HPV-positive or HPV-negative tumor-bearing mice. Overall, no treatment-associated elevation in ALT or AST activity was observed, indicating no detectable hepatocellular injury under the conditions examined (Figure S11). Together, these findings demonstrate localized tumor-growth suppression without detectable treatment-associated injury in the examined major organs or serum hepatic injury markers.

## Discussion

Current therapy for resistant HNSCC remains limited by the difficulty of achieving effective tumor control while avoiding substantial treatment-related morbidity. PDT offers a compelling therapeutic alternative for resistant HNSCC because its phototoxic effects are spatially restricted by both the illuminated field and the tumor selectivity of the photosensitizer. PDT can be integrated with established modalities, including surgery, radiotherapy, and chemotherapy.^3–5^ Yet, despite these advantages, the broader impact of PDT in HNSCC has remained constrained by incomplete and heterogeneous responses, indicating that resistance is driven by more than light delivery, oxygen availability, or PS exposure alone. Here, we used NIR cyanine–carborate PSs as a mechanistic probe to uncover redox-metabolic adaptations that can be targeted to sensitize resistant HNSCC cells to PDT and deliver effective therapy. Our findings identify xCT-dependent cystine utilization as a central mechanism that enables resistant cells to preserve antioxidant buffering during PDT.

PDT response in HNSCC is shaped by tumor-intrinsic redox capacity as well as photophysical parameters. Clinical studies with porfimer sodium and temoporfin have demonstrated meaningful local control in selected head and neck lesions, yet therapeutic responses remain variable even when tumors are technically amenable to treatment.^9,11^ Our data extend this clinical observation by showing that HPV^+^ and HPV^–^ HNSCC models respond differently under otherwise matched photodynamic conditions, as reflected by the markedly lower NIR IC_50_ of SCC2 relative to SCC19 (Figure 1B and Table S2). This divergence is unlikely to reflect HPV status alone and instead points to intrinsic redox-metabolic differences that shape whether PDT-generated ROS is translated into lethal oxidative damage or contained through a nonlethal adaptive response. This interpretation is consistent with clinical and preclinical studies showing that PDT can be effective in HNSCC, yet response heterogeneity remains a major unresolved limitation.^42^

Early adaptive redox remodeling emerges as a central determinant of the resistant phenotype. Although ROS generation is a proximal effector of PDT, sensitive and resistant HNSCC cells appear to differ in their capacity to engage adaptive redox responses following oxidative stress. SCC2 exhibited higher basal total Nrf2 abundance; however, elevated total Nrf2 does not necessarily indicate constitutive pathway activation because Nrf2 transcriptional activity depends on nuclear localization. Accordingly, the early Nrf2-associated transcriptional response following PDT was not accompanied by increased nuclear Nrf2 accumulation, and nuclear Nrf2 levels were reduced at both 2 and 6 h after irradiation (Figure S3). In contrast, resistant SCC19 cells exhibited higher basal ATF4 abundance together with selective xCT induction after PDT (Figure 2C–E). This baseline ATF4 profile may support amino acid and cystine metabolism and facilitate xCT-dependent redox adaptation, although these associations do not independently establish causality. Supportive profiling of additional antioxidant-response genes further suggests that this divergence reflects selective rewiring of downstream redox-defense architecture rather than uniform activation of a single canonical oxidative-stress program (Figure S12). This pattern is consistent with recent work showing that stress-response signaling can couple redox adaptation to cystine utilization and thiol metabolism.^36^ Ferroptosis is an iron-dependent form of regulated cell death driven by lipid peroxidation, making it directly relevant to cystine–glutathione metabolism and PDT-induced oxidative stress. It is also particularly relevant to HNSCC, where recent studies have shown that redox-regulatory pathways, including Nrf2 localization, can shape susceptibility to oxidative stress-associated cell death, including ferroptosis and apoptosis.^43^ Our results further extend prior evidence that therapy resistance in HNSCC is accompanied by metabolic reprogramming toward oxidative stress neutralization rather than simple reduction of oxidative burden.^44^ During PDT, our data support a model in which resistant cells survive through adaptive redox remodeling that limits lipid peroxidation despite ROS induction. This response allows resistant cells to contain oxidative damage before it reaches a lethal threshold.

xCT-dependent cystine utilization functions as a metabolic checkpoint that determines whether PDT is buffered or translated into lethal damage. In resistant SCC19 cells, pharmacologic inhibition of xCT with IKE, extracellular cystine withdrawal, and siRNA-mediated depletion of *SLC7A11* each sensitized cells to PDT, increased lipid peroxidation (Figure 3D,E), and reduced viability across complementary functional assays (Figure 3B–G). These findings indicate that cystine metabolism is functionally required for survival rather than merely associated with resistance. Consistently, *SLC7A11* expression depends on coupling of cystine import to intracellular redox handling,^24^ and ferroptotic susceptibility can be modulated through post-translational stabilization of *SLC7A11*, establishing xCT as a functional gatekeeper of oxidative-stress survival rather than a passive marker of antioxidant state.^45^ Further, thiol-depletion studies show that loss of cystine/GSH supply activates coordinated stress programs that support antioxidant adaptation under redox stress.^46^ In resistant HNSCC cells, ML385-mediated Nrf2 inhibition did not significantly affect viability under PDT stress (Figure S13), indicating that pharmacological inhibition of Nrf2 alone was insufficient to overcome the resistant phenotype under the conditions examined. This negative result does not exclude a contribution from Nrf2, as incomplete pathway inhibition or compensatory stress-response mechanisms may preserve cell survival. In contrast, direct disruption of xCT-dependent cystine utilization through IKE treatment or SLC7A11 knockdown markedly increased PDT phototoxicity, whereas xCT overexpression partially protected PDT-sensitive SCC2 cells (Figures 3B–G and S6). Elevated basal ATF4 abundance in SCC19 may also support SLC7A11 expression through alternative stress-response pathways, although this possibility was not directly tested. Together, these findings indicate that xCT-dependent cystine utilization represents a more direct functional survival dependency than Nrf2 signaling in SCC19 cells under the tested PDT conditions.

Cysteine–glutathione remodeling provides the metabolic basis for adaptive resistance and identifies a therapeutically actionable vulnerability. Whereas sensitive SCC2 cells showed a reduction in intracellular cysteine abundance together with lower GSH/GSSG, NADPH/NADP^+^, and NADH/NAD^+^ ratios, resistant SCC19 cells responded to PDT by increasing cysteine abundance and maintaining a more reduced intracellular state (Figure 4A,B). xCT inhibition reversed this protective phenotype, reducing cysteine levels, decreasing GSH/GSSG and NADPH/NADP^+^ ratios, and sensitizing cells to PDT. Mechanistically, these findings are consistent with the known role of xCT (*SLC7A11*) in importing cystine, which is then reduced to cysteine and used to support GSH synthesis.^47^ This pathway may be particularly relevant for meso-chloro cyanine-based PSs, as these dyes can undergo thiol-mediated substitution by cysteine residues and GSH.^48^ Thus, elevated xCT-dependent cystine metabolism may protect resistant cells by preserving antioxidant capacity while also influencing the chemical fate of meso-chloro cyanine PSs. In support of this model, xCT overexpression in PDT-sensitive SCC2 cells increased tolerance to PDT, supporting xCT as a functional contributor to PDT resistance (Figure S6). These findings are supported by studies showing that the response to xCT-targeted therapy is shaped by metabolic programs that sustain glutamate-linked antioxidant defense in HNSCC cells.^49^ Since both caspase inhibition and Ferrostatin-1 partially rescued cell viability after PDT (Figure S7), our data suggest that xCT disruption engages apoptosis-associated and lipid peroxidation-associated cell death pathways. These results also fit with evidence that disruption of redox-supporting metabolism can increase ferroptotic sensitivity.^50^ *In vivo*, cyanine–carborate PDT in combination with IKE sensitizes HPV^–^ MOE/shPtpn14 tumors to significantly decrease tumor volume (Figure 5C), demonstrating the efficacy of xCT-targeting strategy in a resistant HNSCC model. Together, these findings suggest that resistant cells survive PDT by actively preserving cysteine–glutathione–NADPH homeostasis, thereby limiting ROS-induced oxidative stress and lipid peroxidation. Disrupting this adaptive redox buffering sensitizes PDT-resistant HNSCC models to PDT.

## Conclusion

Our study identifies adaptive redox remodeling as a targetable mechanism that contributes to PDT resistance in HNSCC. Using NIR cyanine–carborate PSs, we show that PDT response is shaped by the capacity of tumor cells to engage xCT-dependent cystine metabolism to support glutathione-linked redox buffering of ROS. Disruption of this pathway collapses redox homeostasis, enhances lipid peroxidation, and sensitizes resistant HPV^–^ HNSCC models to PDT. Together, these findings support a strategy in which redox-targeted interventions are combined with next-generation PSs to improve PDT response in resistant HNSCC and potentially other tumors that rely on cystine–glutathione metabolism during oxidative stress.

## Experimental Section

### Materials

All reagent-grade chemicals, including ethanol, Triton X-100, carboxymethylcellulose sodium and formaldehyde were purchased from Sigma-Aldrich (St. Louis, MO, USA) unless otherwise mentioned. Hoechst 33342, a nucleic acid stain dye, was obtained from Invitrogen (Waltham, MA, USA). CsCB_9_H_10_ and CsCB_11_H_6_Cl were prepared according to literature procedures.^26^

### Synthesis and Purification of Cyanine–Carborates

Cyanine–carborate organic salts were synthesized via ion exchange between the parent cyanine iodide (CyI) and cesium carborate precursors, following previously reported procedures.^26,27^ Briefly, equimolar amounts of cyanine iodide and carborate salts were combined in methanol at room temperature, resulting in rapid precipitation of the corresponding cyanine–carborate salts. The products were isolated by filtration, washed thoroughly to remove residual cesium iodide, and further purified by silica gel filtration using dichloromethane as the eluent. Compound identity and purity were confirmed by UHPLC high-resolution mass spectrometry in both positive and negative ion modes, verifying the presence of the cyanine cation and carborate counterion, respectively.^26^

### Cell Culture

Human head and neck squamous cell carcinoma cells, HPV^+^ (SCC2, SCC90, and SCC152) and HPV^–^ (SCC1, SCC4, and SCC19), were cultured in an incubator at 37 °C and 5% CO_2_ in growth Dulbecco’s Modified Eagle’s Medium (Cat. No. 10–017CM, Corning, NY, USA) supplemented with 10% heat-inactivated fetal bovine serum (Cat. No. F0392, Sigma, St. Louis, MO, USA), 2 mM glutamine, 100 U/mL penicillin, and 100 μg/mL streptomycin (Cat. No. 15323671, Corning, NY, USA).^17^ Mouse head and neck squamous cell carcinoma cell lines MOE/shPtpn14 (HPV^–^) and mEERL (HPV^+^) were cultured under the same conditions, as previously described.^31^

### Cyanine–Carborate Treatment

Fluorescent cyanine-carborates were dissolved to form a 2 mM in dimethyl sulfoxide (Cat. No. D4540, Millipore Sigma, St. Louis, MO, USA), and then diluted in Dulbecco’s Modified Eagle’s Medium to flash nanoprecipitate a stable colloidal nanoparticle suspension.

### Cell Viability

Human and mouse HNSCC cells were seeded in 6-well tissue culture plates at a density of 100,000 cells per well and treated with cyanine–carborate PSs at the indicated concentrations. After 24 h, the treatment medium was removed and replaced with fresh growth medium without PS to minimize extracellular phototoxicity. Cells were then exposed to an 850 nm LED light source at an irradiance of 425 mW/cm^2^ for 30 min under standard culture conditions, while control cells were maintained in the dark. Thus, PDT under these conditions was mediated primarily by cell-internalized PSs. Following irradiation, fresh medium containing PSs was added, and cells were incubated for an additional 24 h. This photodynamic treatment was performed a total of two times (at 24 and 48 h). Cell viability was assessed on day 3 by trypan blue exclusion using an automated cell counter (Cellometer Auto T4, Nexcelom Bioscience, MA, USA). In selected experiments, cell viability was independently confirmed using the Alamar Blue (Cat. No. DAL125, Thermo Scientific) assay according to the manufacturer’s instructions to validate metabolic activity–based measurements. All experiments were performed with three biological replicates.

### Colocalization Analysis

HNSCC cells were seeded onto 20 mm glass-bottom dishes (Cat. No. 801001, Nest Biotechnology) and allowed to adhere for 24 h under standard culture conditions. Cells were then treated with cyanine–carborate PSs at a final concentration of 1 μM and incubated for an additional 24 h. To assess subcellular localization, cells were separately stained with Rhodamine 123 (Cat. No. R302, Thermo Scientific), LysoTracker Green (Cat. No. L7526, Thermo Scientific), ER-Tracker Green (Cat. No. E34251, Thermo Scientific), or Golgi-specific dye (Cat. No. D7450, Thermo Scientific) following the manufacturers’ protocols. After staining, cells were rinsed three times with phosphate-buffered saline (PBS) prior to imaging. Fluorescence images were acquired using a 63× oil-immersion objective. Organelle-specific probes were excited and detected using the recommended filter settings, while cyanine–carborate fluorescence was visualized using a custom filter set (excitation 350/50 nm, emission 650 nm long-pass) mounted in a Leica DMi8 microscope (Leica Microsystems) and manufactured by Chroma Technology Corp.

### Measuring Reactive Oxygen Species (ROS)

Cells were seeded onto 20 mm glass-bottom dishes and cultured under standard conditions with or without cyanine–carborate PSs at a final concentration of 1 μM. After 24 h of incubation, PS-treated cells were exposed to an 850 nm LED light source for 30 min in the incubator, while dark control cells were maintained without irradiation. Immediately following light exposure, intracellular reactive oxygen species were detected using chloromethyl-2′,7′-dichlorodihydrofluorescein diacetate (CM-H_2_DCFDA; Cat. No. C6827, Invitrogen), MitoSOX Red (Cat. No. M36006, Invitrogen), or Singlet Oxygen Sensor Green (Cat. No. S36002, Invitrogen), applied according to the manufacturers’ protocols. Cells were then washed three times with phosphate-buffered saline (PBS) prior to imaging. Fluorescence images were acquired using a 40× objective lens under identical acquisition settings across all conditions.

### Lipid Peroxidation Imaging

Lipid peroxidation was assessed in live cells using the Image-iT Lipid Peroxidation Kit (Cat. No. C10445, Thermo Fisher Scientific). HNSCC cells were seeded for 24 h and treated with cyanine–carborate PSs as indicated. Following overnight incubation, cells were irradiated with an 850 nm LED light source for 25 min, while dark controls were not exposed to light. Cells were then incubated with 10 μM Image-iT Lipid Peroxidation Sensor reagent for 30 min at 37 °C in the dark, washed once with PBS, and imaged live. Fluorescence images were acquired using a Leica DMi8 microscope with a 40× objective. Oxidized and nonoxidized sensor signals were detected in red and green channels, respectively, and lipid peroxidation was quantified as the ratio of red to green fluorescence intensity. All experiments were performed with at least three biological replicates.

### Cell Death Inhibition Studies

HNSCC cells were treated with pharmacologic inhibitors to assess the contribution of regulated cell death pathways following PDT. Ferrostatin-1 (2 μM) was used to inhibit ferroptosis, z-VAD-fmk (50 μM) to block apoptosis, Necrostatin-1 (10 μM) to inhibit necroptosis, and bafilomycin A1 (10 nM) to assess autophagic flux. All inhibitors were added 2 h prior to PDT and maintained throughout the postirradiation period. Cell viability was assessed 24 h after NIR irradiation.

### Nuclear Factor Erythroid 2-Related Factor 2 (Nrf2) Inhibition

For Nrf2 inhibition experiments, SCC19 cells were treated with the Nrf2 inhibitor ML385 (Cat. No. S8790, Selleckchem) at the indicated concentrations before PDT. Cells were maintained in ML385-containing medium during the postirradiation period, and viability was assessed after 48 h.

### xCT (*SLC7A11*) Knockdown

SCC19 cells were transfected with small interfering RNA (siRNA) targeting xCT (*SLC7A11*) or a nontargeting control siRNA using Lipofectamine 3000 (Thermo Fisher Scientific) according to the manufacturer’s instructions. Cells were seeded 24 h prior to transfection to achieve ∼50–70% confluency at the time of transfection. siRNA–lipid complexes were prepared in Opti-MEM and added to cells in antibiotic-free medium. After 6 h, the transfection medium was replaced with fresh complete growth medium, and cells were incubated for 48–72 h prior to downstream analyses. Knockdown efficiency was confirmed by qPCR and immunoblotting. siRNA targeting *SLC7A11* and nontargeting control siRNA were obtained from Integrated DNA Technologies (IDT, USA).

### xCT (*SLC7A11*) Overexpression

SCC2 cells were transfected with a human xCT (*SLC7A11*) expression plasmid obtained from VectorBuilder (Vector ID: VB260317–1377TTK) or the corresponding empty vector control using Lipofectamine 3000 according to the manufacturer’s instructions. Cells were seeded 24 h before transfection to achieve approximately 50–70% confluency at the time of transfection. After transfection, cells were incubated for 48 h before PDT treatment or immunoblot analysis. xCT (*SLC7A11*) overexpression was confirmed by Western blotting (Figure S6).

### Liquid Chromatography–Mass Spectrometry (LC-MS)

HNSCC cells were seeded in six-well plates and subjected to the indicated treatments and PDT conditions. Cellular metabolism was quenched directly on the plate by sequential addition of 200 μL methanol containing 100 mM *N*-ethylmaleimide (NEM), followed immediately by 200 μL methanol containing isotopically labeled internal standards. Cells were scraped into prechilled microcentrifuge tubes, vortexed vigorously for 5 min, and incubated for an additional 45 min at room temperature to allow complete thiol derivatization. Samples were then centrifuged at 4 °C for 10 min at maximum speed, and 300 μL of the supernatant was transferred to a fresh tube and dried using a benchtop freeze-dryer for 24 h (Labconco, MO, USA). Dried metabolite extracts were reconstituted in 200 μL of 10% PFHA and transferred to LC autosampler vials with low-volume glass inserts for analysis. ^13^C/^15^N-labeled amino acids were included as internal standards for all metabolites, and the remaining protein pellet was retained for normalization. Reduced glutathione (GSH), oxidized glutathione (GSSG), cysteine, glutamate, and related metabolites were quantified based on peak areas normalized to their respective isotopically labeled internal standards and total protein content. Peak integration and data processing were performed using MAVEN.^32^ For NADPH analysis, metabolites were extracted separately in acetonitrile:methanol:water (40:40:20, v/v/v). Cells were scraped into extraction solvent, incubated on ice for 30 min, supplemented with 15% NH_4_HCO_3_, and incubated for an additional 20 min on ice. Samples were then centrifuged at 16,000*g* for 15 min at 4 °C, and the supernatants were collected for QTOF analysis. Statistical analyses were conducted in R using Student’s *t* test with Benjamini–Hochberg correction for multiple comparisons where appropriate.

### Quantitative Polymerase Chain Reaction (qPCR) Analysis

Total RNA was isolated from HNSCC cells at 2 and 6 h following PDT using the RNeasy Plus Micro Kit (Cat. No. 74034, Qiagen, MD, USA) according to the manufacturer’s instructions. Extracted RNA (1 μg) was reverse transcribed into cDNA using a 2× RT master mix (Cat. No. 4368814, Thermo Fisher Scientific). Quantitative PCR was performed using Luna Universal qPCR Master Mix (2×) (Cat. No. M3003S, New England Biolabs) on a real-time PCR system (Agilent, USA). Primer sequences used for gene expression analysis are listed in Table S1.

### Western Blotting

Whole-cell protein lysates were prepared using Cell Lysis Buffer (10×) (Cat. No. 9803, Cell Signaling Technology) supplemented with protease and phosphatase inhibitors. For nuclear and cytoplasmic protein analysis, cells were fractionated using the NE-PER Nuclear and Cytoplasmic Extraction Kit (Cat. No. 78833, Thermo Fisher Scientific) according to the manufacturer’s instructions. Protein concentrations were determined using a BCA assay. Equal amounts of protein (50 μg) were separated on 10% or 12% SDS–polyacrylamide gels and electrotransferred onto nitrocellulose membranes. Membranes were washed with Tris-buffered saline (10 mM Tris-HCl, 150 mM NaCl, pH 7.5) containing 0.05% (v/v) Tween-20 (TBST) and blocked with TBST supplemented with 5% (w/v) nonfat dry milk for 1 h at room temperature. Membranes were incubated overnight at 4 °C with primary antibodies against Nrf2 (Cat. No. 12721, Cell Signaling Technology, 1:1000), Keap1 (Cat. No. 8047, Cell Signaling Technology, 1:1000), ATF4 (Cat. No. 11815S, Cell Signaling Technology, 1:1000), xCT (*SLC7A11*) (Cat. No. 12691, Cell Signaling Technology, 1:1000), HMOX-1 (Cat. No. 5061, Cell Signaling Technology, 1:1000), β-actin (Cat. No. 4970, Cell Signaling Technology, 1:5000), and histone H3 (Cat. No. 4499, Cell Signaling Technology, 1:2000). β-actin and histone H3 were used as loading controls for cytoplasmic and nuclear fractions, respectively. After washing with TBST, membranes were incubated with horseradish peroxidase (HRP)–conjugated secondary antibodies for 1 h at room temperature. Protein bands were detected using enhanced chemiluminescence (ECL) reagents and visualized using a Bio-Rad chemiluminescent imaging system (Hercules, CA, USA).

### Flank Tumor Model

All animal studies were approved by and conducted in accordance with the guidelines of the Institutional Animal Care and Use Committee (IACUC) of Michigan State University (protocol number: 202500130). Male albino C57BL/6 mice (Jackson Laboratory strain 000058) aged 6–8 weeks were used for all experiments. For tumor implantation, mouse HNSCC cell lines MOE/shPtpn14 (HPV^–^) or mEERL (HPV^+^) were cultured to approximately 80% confluency and harvested during the logarithmic growth phase. Cells were resuspended in sterile saline, and 5 × 10^5^ cells in a total volume of 50 μL were injected subcutaneously into the flank of each mouse. Tumor growth was monitored every other day using digital calipers, and tumor volume was calculated using the formula

$$V=\left(L\times W^{2}\right)/2$$

where *L* represents tumor length and *W* represents tumor width. Animal health was assessed throughout the study by monitoring body weight and general condition, including inspection of the tumor site for signs of ulceration or irritation. Mice were euthanized at experimental end points or earlier if humane end point criteria were met due to tumor burden.

### *In Vivo* Photodynamic Therapy (PDT)

When flank tumors reached the appropriate size, approximately 9 days after tumor-cell implantation, mice were randomized into the treatment groups indicated for each tumor model. Mice assigned to photosensitizer treatment received the cyanine–carborate PS at 2.5 μmol/kg by intravenous injection through the lateral tail vein. The PSs were first dissolved in 50 μL of DMSO and then diluted with 950 μL of a solution containing 0.03% Tween 20 and 10% DMSO. The treatment schedule consisted of two photosensitizer administration cycles. The photosensitizer or vehicle was administered on treatment days 0 and 6. 24 h after each administration, mice assigned to PDT were anesthetized with 2.5% isoflurane, placed on a temperature-controlled heating pad, and the tumors were irradiated with an 850 nm LED light source at a total light dose of 150 J/cm^2^ delivered over 15 min. Subsequent irradiation sessions were performed at 48-h intervals. Accordingly, NIR irradiation was administered on treatment days 1, 3, 5, 7, 9, and 11, for a total of six irradiation sessions. For studies involving xCT inhibition in HPV-negative tumor-bearing mice, imidazole ketone erastin (IKE) was administered by oral gavage at 50 mg/kg once daily for 14 consecutive days and was formulated in phosphate-buffered saline containing 0.5% carboxymethylcellulose sodium and 0.1% Tween 80. On each PDT treatment day, IKE was administered 2 h before NIR irradiation, whereas on intervening days without PDT, it was administered once daily according to the same dosing schedule. The 2-h interval between IKE administration and irradiation was maintained throughout the combination-treatment study. Fluorescence imaging was performed at designated time points after photosensitizer administration to assess photosensitizer distribution and tumor localization. Mice were euthanized on the 12th day of treatment, and tumors and major organs were collected for subsequent biochemical, histological, and imaging analyses.

### *In Vivo* Imaging

For biodistribution analysis, tumor-bearing mice were administered cyanine–carborate PSs (CyCB_11_H_6_Cl_6_ or CyCB_9_HCl_9_) at a dose of 2.5 μmol/kg via intravenous injection through the lateral tail vein when flank tumors were established. Prior to imaging, mice were anesthetized using 2.5% isoflurane. Brightfield and near-infrared (NIR) fluorescence images were acquired at designated time points using a Leica M165 FC stereomicroscope equipped with a 740 nm PE4000 LED light source and a DFC9000 GT camera. Fluorescence images were analyzed using Leica imaging software to quantify signal intensity within defined regions of interest (ROIs), including the tumor, liver, and surrounding nontumor tissue. Fluorescence intensities were normalized to values obtained from vehicle-injected control mice to account for background signal.

### Histology and Hematoxylin and Eosin (H&E) Staining

Mouse tissues (lung, kidney, liver, spleen, heart, and tumor) were collected at experimental end points, fixed in 10% neutral-buffered formalin, paraffin-embedded, and sectioned at 5 μm. Sections were deparaffinized, rehydrated, stained with H&E, dehydrated, cleared, and mounted. Stained slides were imaged by brightfield microscopy to assess tissue morphology.

### Immunohistochemistry (IHC)

Immunohistochemical staining was performed for Nrf2, xCT, and ATF4. Antigen retrieval conditions were optimized separately for each target. For Nrf2 staining, sections underwent heat-induced antigen retrieval in pH 6.0 retrieval buffer using a steamer. For xCT staining, antigen retrieval was performed in pH 9.0 retrieval buffer using a pressure cooker. For ATF4 staining, sections underwent pressure-cooker antigen retrieval in pH 6.0 retrieval buffer. Following antigen retrieval, sections were incubated with RBM for 20 min and then incubated overnight at 4 °C with primary antibodies against Nrf2, xCT, or ATF4 at dilutions of 1:400, 1:500, and 1:200, respectively. Sections were subsequently incubated with an HRP-conjugated detection reagent for 30 min. Immunoreactivity was visualized using 3-amino-9-ethylcarbazole (AEC) chromogen for 5 min. Nrf2- and xCT-stained sections were counterstained with hematoxylin diluted 1:10 for 1 min, whereas ATF4-stained sections were counterstained with hematoxylin diluted 1:5 for 3 min.

### Serum Alanine Aminotransferase (ALT) and Aspartate Aminotransferase (AST) Activity Assays

At the experimental end point, whole blood was collected from tumor-bearing mice by cardiac puncture and allowed to clot at room temperature. Samples were centrifuged to isolate serum, and the serum fraction was stored at −80 °C until analysis. ALT (Sigma-Aldrich, Cat. No. MAK052) and AST (Sigma-Aldrich, Cat. No. MAK055) activities were measured using commercially available assay kits according to the manufacturers’ instructions. Values from HPV-negative samples were normalized to the mean of the corresponding HPV-negative dark-control group, whereas values from HPV-positive samples were normalized to the mean of the corresponding HPV-positive dark-control group. Results are presented as the percentage of the respective control groups.

### Statistical Analyses

Statistical analyses were performed using GraphPad Prism. Two-group comparisons were analyzed using unpaired two-tailed Student’s *t* tests. Multigroup comparisons were analyzed using one-way or two-way ANOVA with appropriate posthoc multiple comparison corrections. Tumor growth curves were analyzed using two-way repeated-measures ANOVA. Data are presented as mean ± SD unless otherwise indicated.

## Supplementary Material



## Notes

Description of photodynamic activity, cellular uptake, and subcellular localization of cyanine–carborate photosensitizers in HNSCC cells; Nrf2 and xCT responses following PDT; effects of cystine withdrawal, xCT overexpression, IKE treatment, and pathway-directed cell-death inhibitors on PDT response; LC–MS analysis of redox-associated metabolites; *in vivo* photosensitizer retention, body-weight monitoring, organ distribution, and toxicity assessment; immunohistochemical and antioxidant-response analyses in HPV-positive and HPV-negative HNSCC models; Nrf2 inhibition studies; qPCR primer sequences; cyanine–carborate IC_50_ values; and quantitative histopathological scoring of tumors and major organs (PDF)
