# Supplementary material for: Cystine-Dependent Redox Metabolism Determines Photodynamic Therapy Response in Head and Neck Squamous Cell Carcinoma
Source: ACS Nano. 2026 Sep 7;20(37):25531–46. doi: 10.1021/acsnano.6c10240 (PMC13602999; doi:10.1021/acsnano.6c10240)
Supplement: Supplementary file 1 [file nn6c10240_si_001.pdf]

Supporting information for

Cystine-dependent Redox Metabolism Determines Photodynamic Therapy Response in Head and Neck Squamous Cell Carcinoma

Amir Roshanzadeh [a], Hyllana C. D. Medeiros [a], Tessa Jordan [a], Carson Malhado [b], Katherine Espinoza Morelos [c], Mohamed Khalil [d,e], Jeyashri Muthukumar [b], Laura Favazza [f], Steven S. Chang [g,h], James M. Hamby [i], Laura C. Cesa [i], Dohun Pyeon [d,*], Vincent Lavallo [c,*], Richard R. Lunt [b, j,*], Sophia Y. Lunt [a, b,*]

[a] Department of Biochemistry and Molecular Biology, Michigan State University, East Lansing, MI, 48824, United States.

[b] Department of Chemical Engineering and Materials Science, Michigan State University, East Lansing, MI, 48824, United States.

[c] Department of Chemistry, University of California, Riverside, CA, 92521, United States.

[d] Department of Microbiology, Genetics, and Immunology, Michigan State University, East Lansing, MI, 48824, United States.

[e] Department of Molecular Biology, National Research Center, Cairo, Egypt.

[f] Department of Pathology and Laboratory Medicine, Henry Ford Health, Detroit, MI, 48202, United States.

[g] Department of Otolaryngology–Head and Neck Surgery, College of Human Medicine, Michigan State University, East Lansing, MI, 48824, United States.

[h] Department of Otolaryngology–Head and Neck Surgery, Henry Ford Health, 2799 west grand blvd, K-building 8th floor, Detroit, MI, 48202, United States.

[i] Entrepreneur-in-Residence (EIR) Spartan Innovations, Michigan State University, East Lansing, MI, 48824, United States.

[j] Department of Physics and Astronomy, Michigan State University, East Lansing, MI, 48824, United States.

Supporting Information Includes:

Figures S1–S13

Figure S1. Photodynamic activity of cyanine-carborate photosensitizers in HNSCC.

Figure S2. Cellular uptake and subcellular localization of cyanine–carborate photosensitizers in HNSCC cells.

Figure S3. Nuclear Nrf2 response following PDT in PDT-sensitive SCC2 cells.

Figure S4. Effect of cystine withdrawal on viability in PDT-sensitive and PDT-resistant HNSCC cells.

Figure S5. xCT induction and IKE-mediated sensitization in additional HNSCC models.

Figure S6. xCT overexpression partially protects PDT-sensitive SCC2 cells from photodynamic therapy.

Figure S7. Effects of pathway-directed cell-death inhibitors on SCC19 viability following photodynamic therapy.

Figure S8. Individual LC–MS measurements of redox-associated metabolites in SCC2 and SCC19 cells following photodynamic therapy.

Figure S9. Extended *in vivo* photosensitizer retention and body weight monitoring following cyanine–carborate PDT.

Figure S10. Immunohistochemical detection of ATF4, Nrf2, and xCT in HPV-positive and HPV-negative HNSCC tumors.

Figure S11. Serum ALT and AST activities following PDT in HPV-positive and HPV-negative HNSCC tumor-bearing mice.

Figure S12. Differential antioxidant-response genes in PDT-sensitive and PDT-resistant HNSCC cells.

Figure S13. Nrf2 inhibition does not substantially sensitize PDT-resistant SCC19 cells to photodynamic stress.

Tables S1–S3

Table S1. Sequences of primers used for real-time quantitative PCR.

Table S2. Half maximal inhibitory concentrations (IC_{50}) of cyanine-carborates with and without NIR irradiation in head and neck squamous cell carcinoma cell lines

Table S3. Quantitative histopathological scoring of tumor and major-organ necrosis following cyanine–carborate photodynamic therapy.

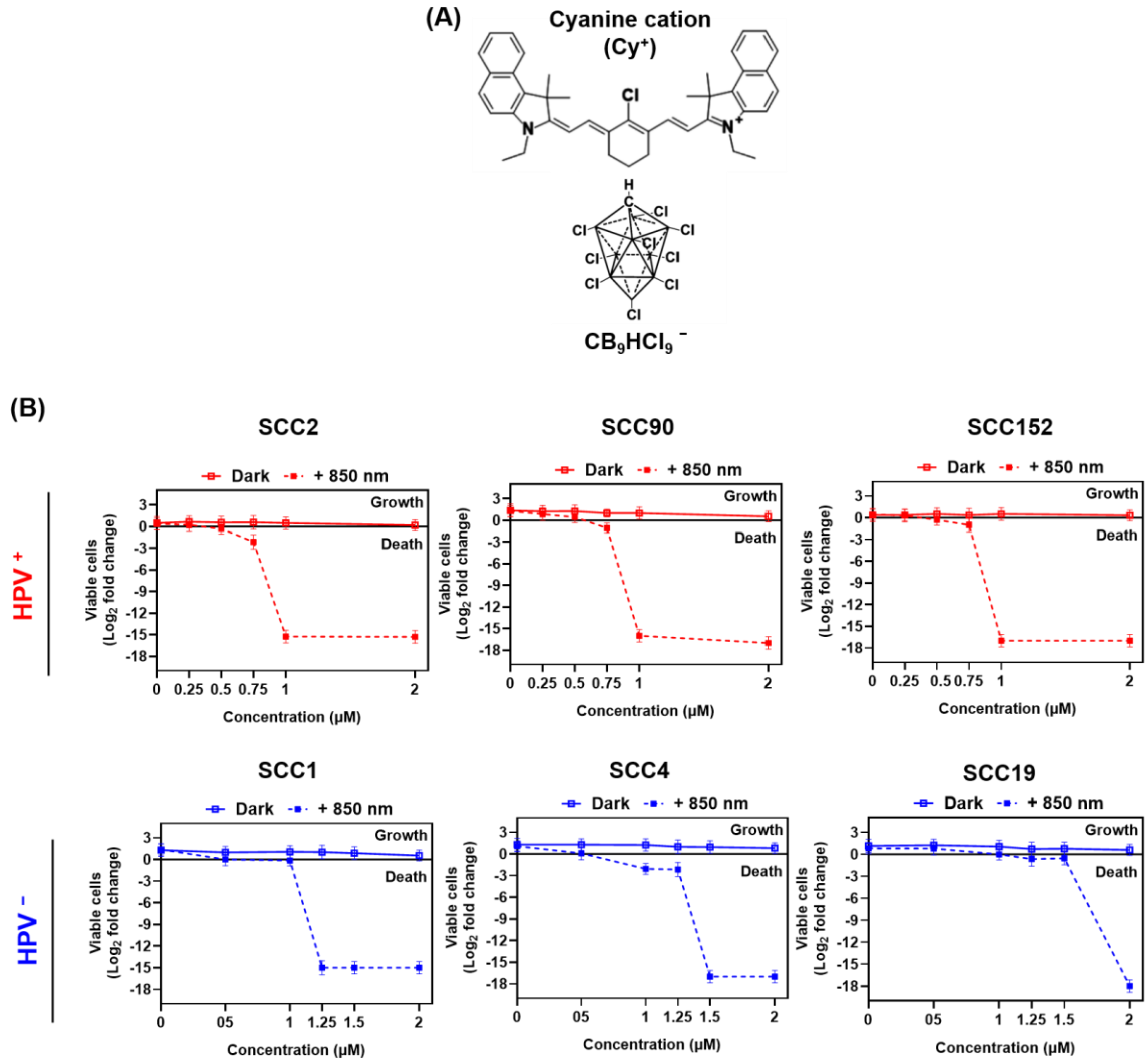

Figure S1. Photodynamic activity of cyanine-carborate photosensitizers in HNSCC. (A) Chemical structures of the cyanine cation paired with $CB_9HCl_9^-$ carborate counterions used as near-infrared (NIR) photosensitizers. (B) Concentration–response analysis of cell viability following cyanine–carborate treatment under dark conditions or NIR (850 nm) irradiation in HPV-positive (SCC2, SCC90, SCC152) and HPV-negative (SCC1, SCC4, SCC19) HNSCC cell lines.

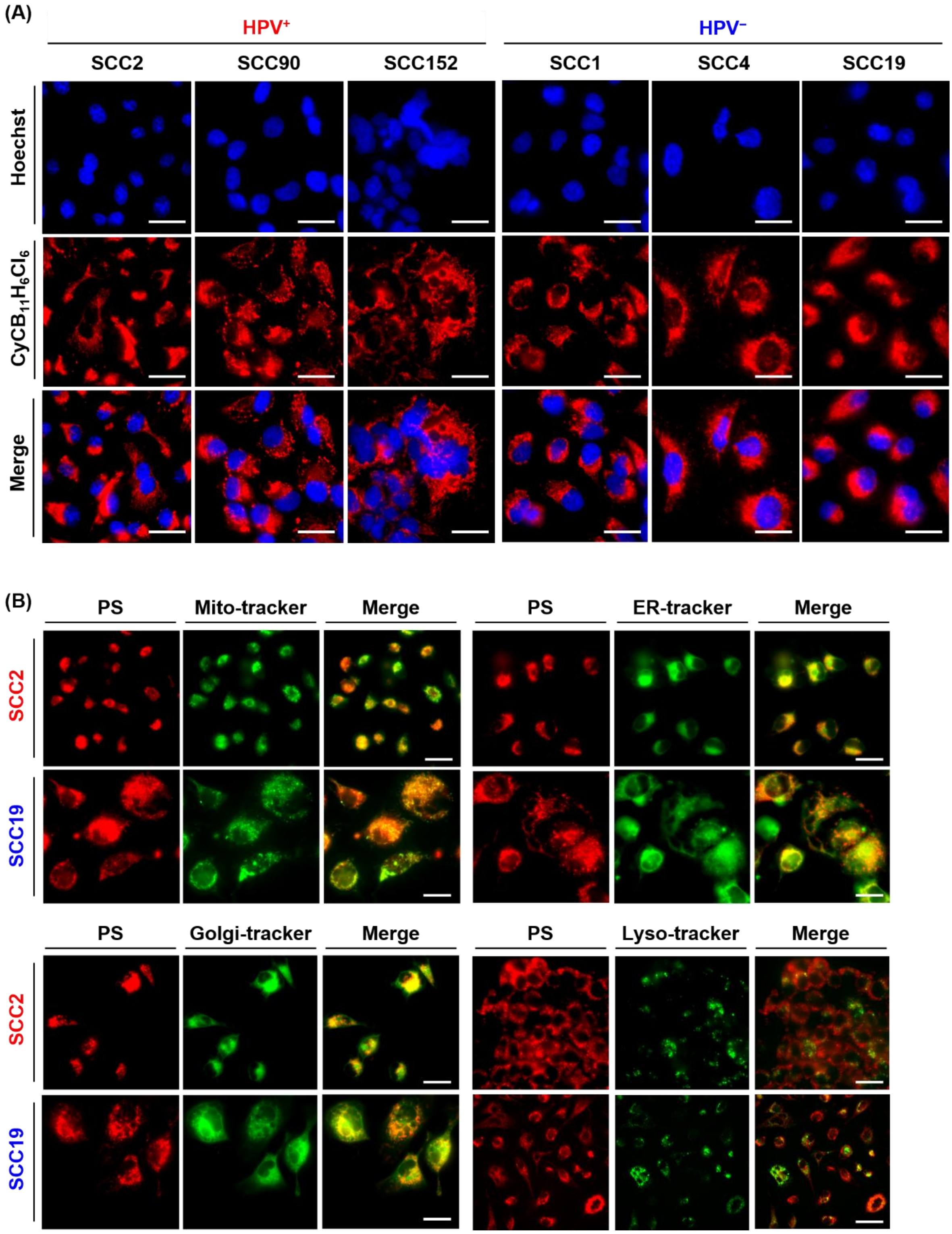

Figure S2. Cellular uptake and subcellular localization of cyanine–carborate photosensitizers in HNSCC cells. (A) Representative fluorescence images showing cellular uptake of $CyCB_{11}H_6Cl_6$ in HPV^+ (SCC2, SCC90, SCC152) and HPV^- (SCC1, SCC4, SCC19) HNSCC cell lines. Nuclei were stained with Hoechst 33342, and cyanine–carborate fluorescence is shown in red. Merged images demonstrate intracellular accumulation across both HPV-positive and HPV-negative models. Scale bars: 70 µm. (B) Subcellular colocalization analysis

of cyanine–carborate photosensitizer fluorescence in representative PDT-sensitive SCC2 and PDT-resistant SCC19 cells. Cells were co-stained with organelle-specific trackers for mitochondria, endoplasmic reticulum, Golgi apparatus, and lysosomes. Scale bars: 70 μm.

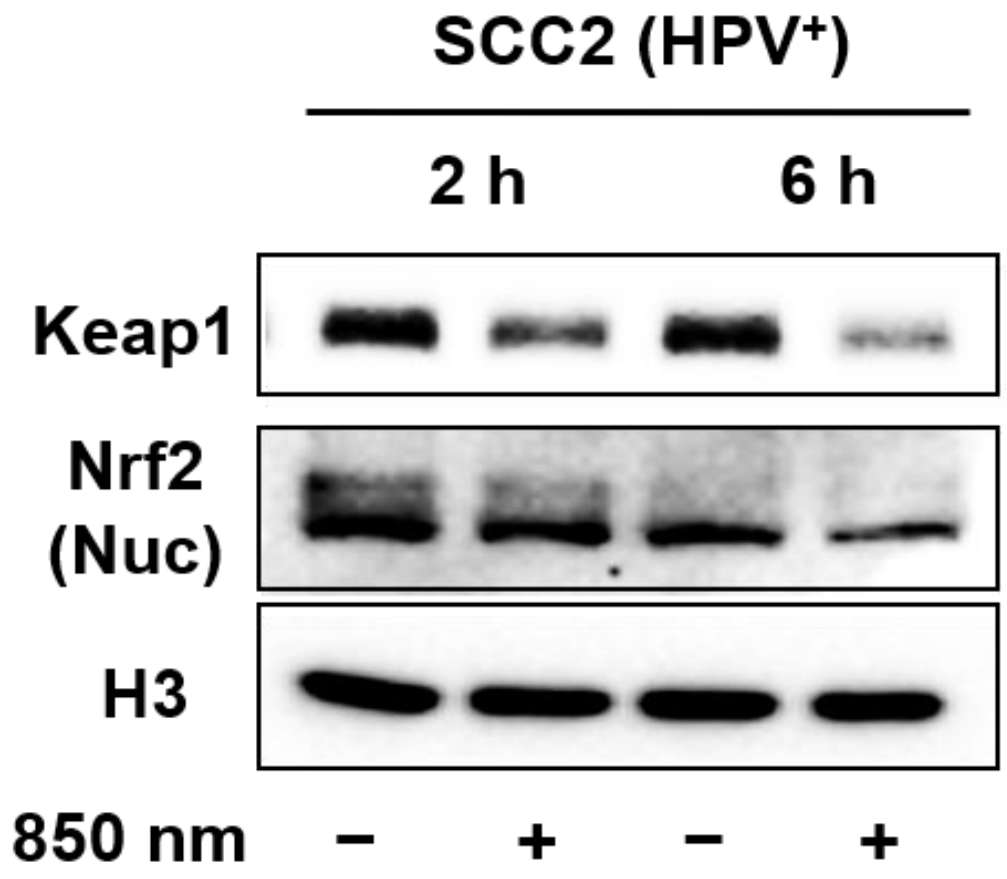

Figure S3. Nuclear Nrf2 response following PDT in PDT-sensitive SCC2 cells. Nuclear fractionation immunoblot analysis of KEAP1 and nuclear Nrf2 in PDT-sensitive SCC2 cells at 2 h and 6 h following NIR irradiation. Histone H3 was used as a nuclear loading control.

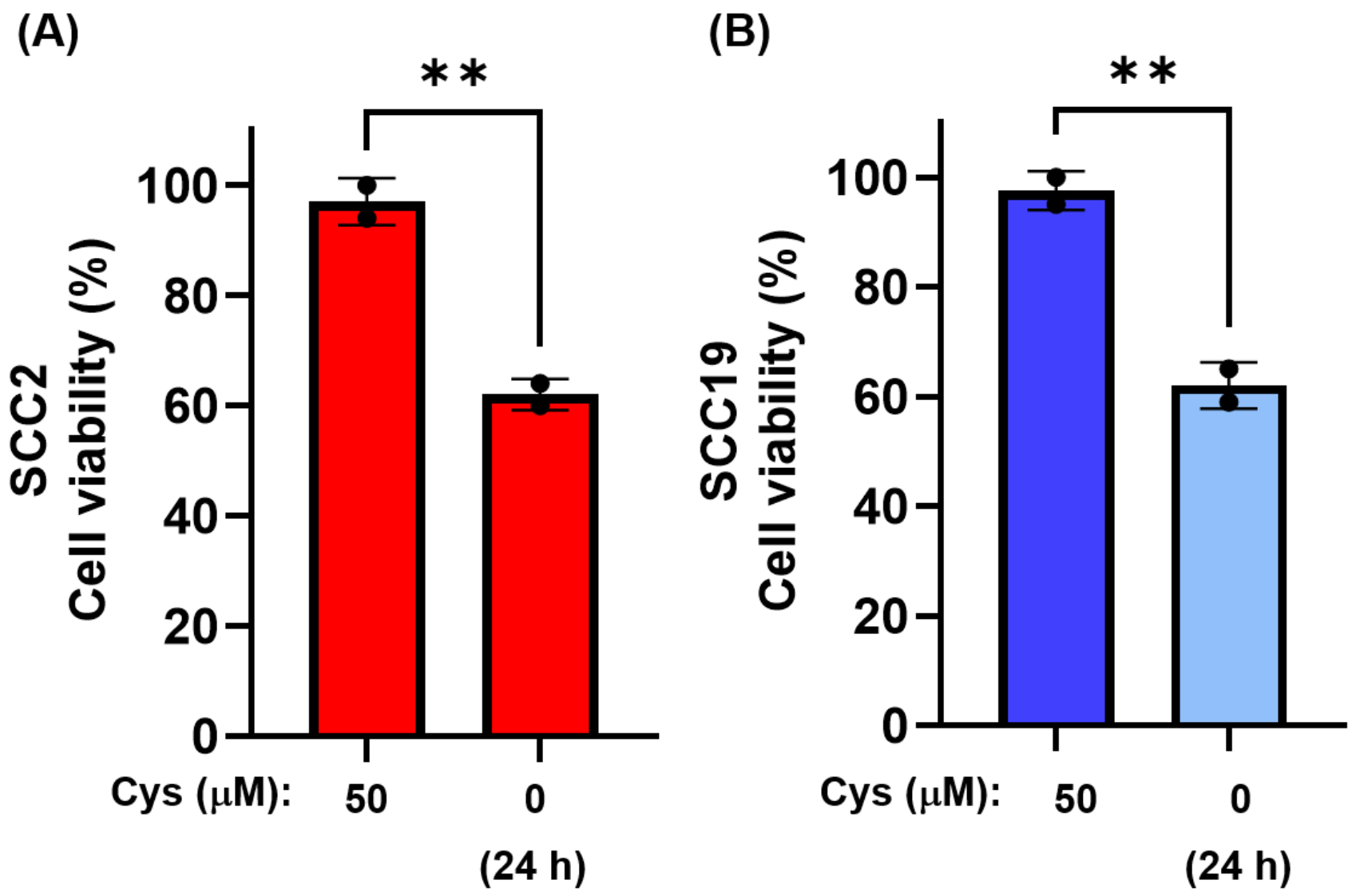

Figure S4. Effect of cystine withdrawal on viability in PDT-sensitive and PDT-resistant HNSCC cells. (A) PDT-sensitive SCC2 and (B) PDT-resistant SCC19 cells were cultured under physiologic-range cystine conditions (50 µM) or cystine-free conditions. Cell viability was measured after 24 h of cystine starvation using the Alamar Blue assay. Data are presented as mean ± S.D.

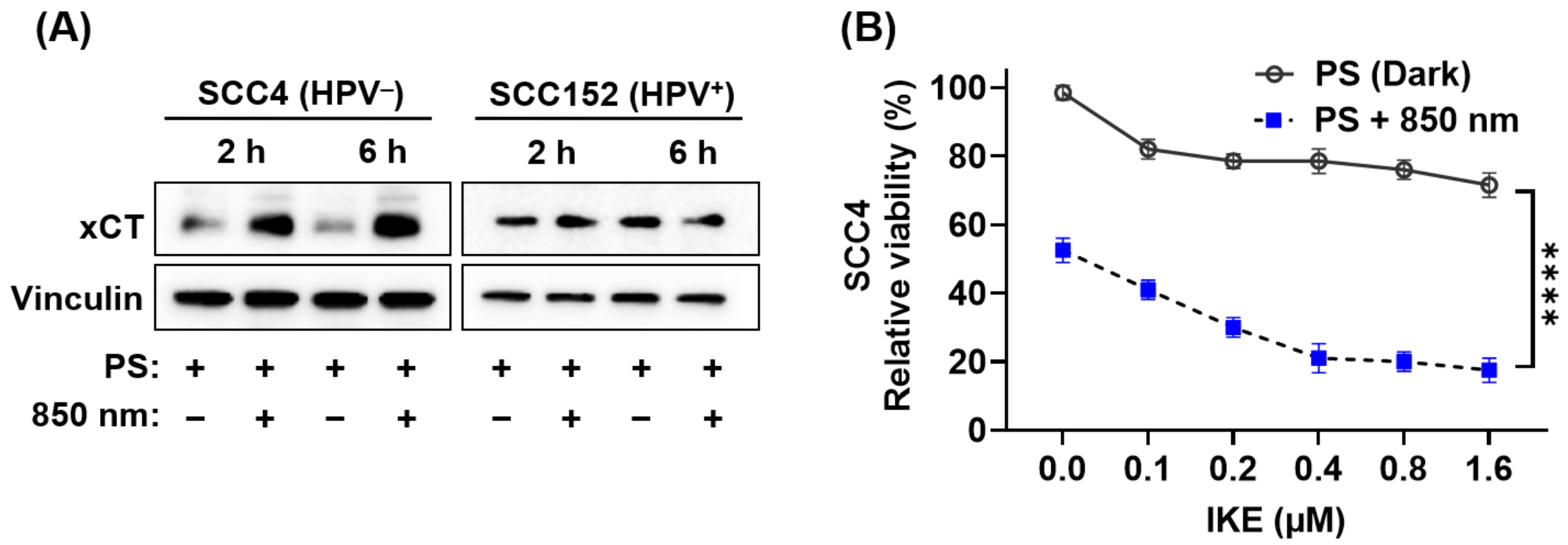

Figure S5. xCT induction and IKE-mediated sensitization in additional HNSCC models. (A) Immunoblot analysis of xCT expression in SCC4 (HPV^{-}) and SCC152 (HPV^{+}) cells at 2 h and 6 h following PDT. Vinculin was used as a loading control. (B) Viability of SCC4 cells treated with increasing concentrations of imidazole ketone erastin (IKE) under dark conditions or following NIR irradiation. IKE treatment enhanced sensitivity to PDT in SCC4 cells, supporting xCT-dependent cystine metabolism as a resistance-associated vulnerability in HPV− HNSCC. Data are presented as mean ± S.D.

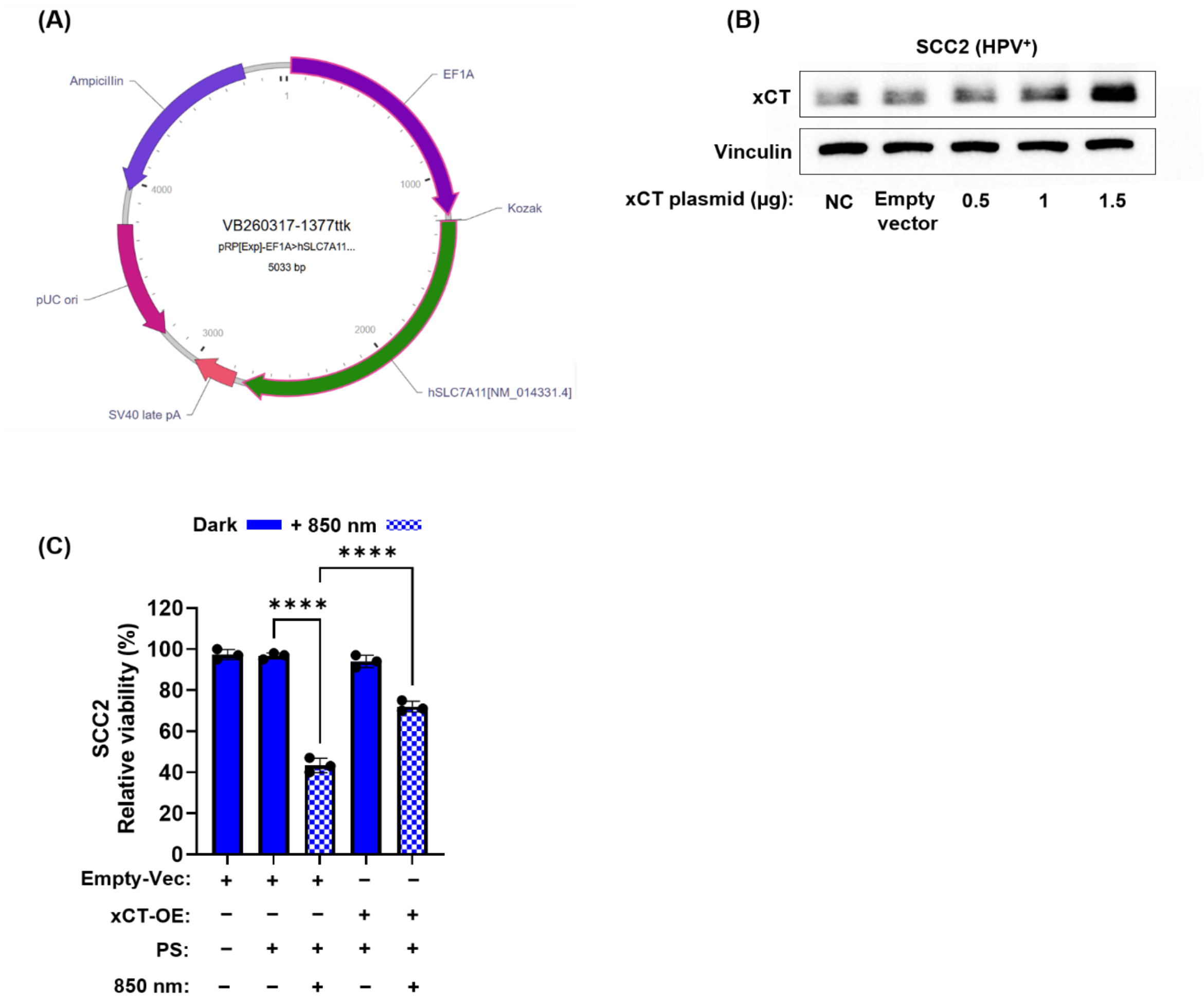

Figure S6. xCT overexpression partially protects PDT-sensitive SCC2 cells from photodynamic therapy. (A) Schematic of the SLC7A11/xCT overexpression plasmid used to ectopically express human xCT. (B) Immunoblot validation of xCT protein expression following transfection in SCC2 cells. Vinculin was used as a loading control. (C) Viability of control and xCT-overexpressing SCC2 cells under dark conditions or following PDT. xCT overexpression partially rescued SCC2 cell viability after NIR irradiation, indicating that increased xCT expression enhances resistance to photodynamic stress. Data are presented as mean ± S.D.

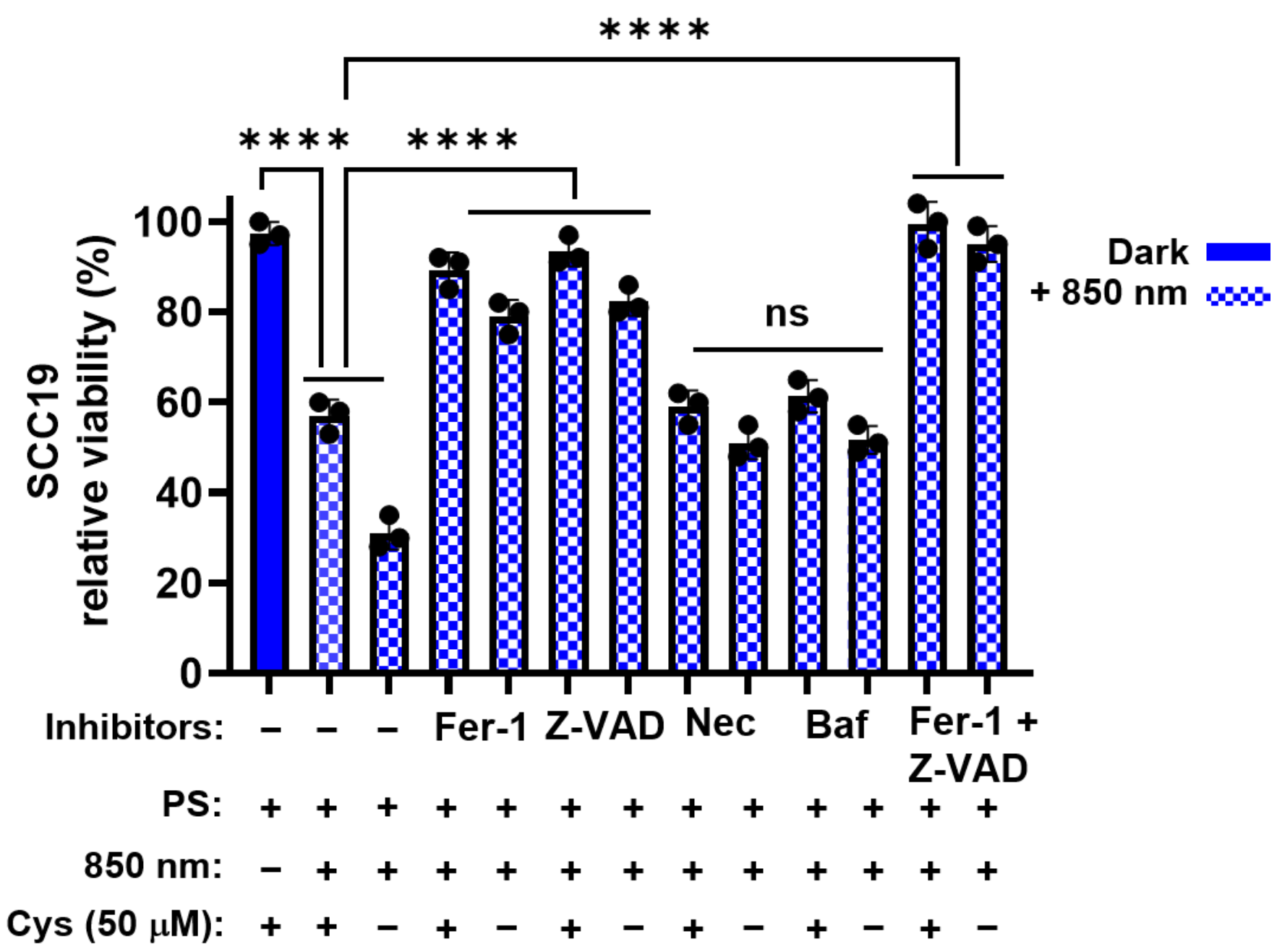

Figure S7. Effects of pathway-directed cell-death inhibitors on SCC19 viability following photodynamic therapy. Viability of SCC19 cells following PDT in the presence of ferrostatin-1 (Fer-1), necrostatin-1 (Nec), bafilomycin A1 (Baf), or the pan-caspase inhibitor z-VAD-fmk (zVAD), treated individually or in combination as indicated. Cell viability was measured 24 h after PDT in the presence of inhibitors using the Alamar Blue assay. Data are presented as mean ± S.D. (n = 3; **P* < 0.05, ***P* < 0.01, ****P* < 0.001; ns, not significant).

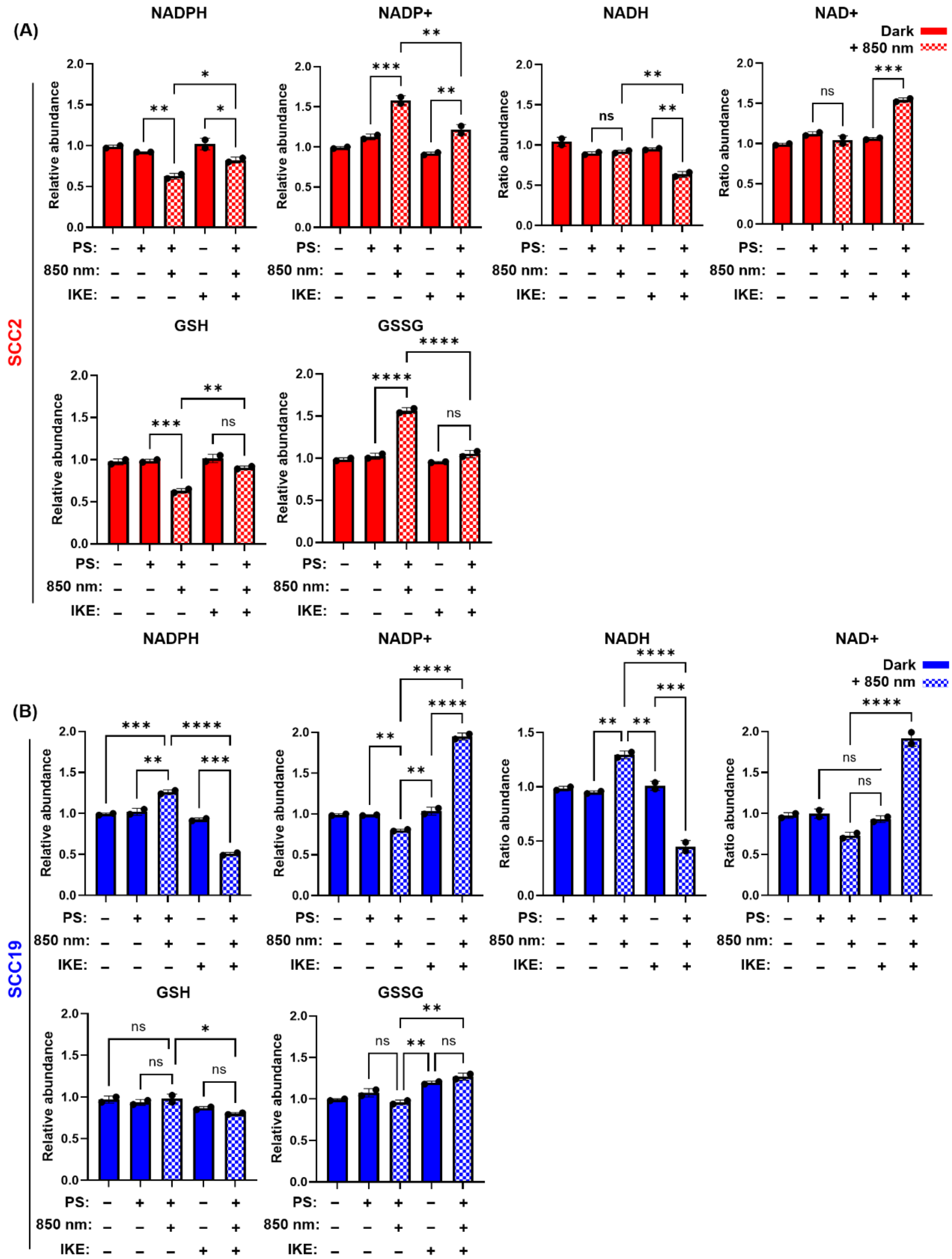

Figure S8. Individual LC–MS measurements of redox-associated metabolites in SCC2 and SCC19 cells following photodynamic therapy. Relative abundances of NADPH, $NADP^+$, NADH, NAD^+, GSH, and GSSG were measured by LC–MS in (A) PDT-sensitive SCC2 cells and (B) PDT-resistant SCC19 cells under control, photosensitizer-only dark, PDT, IKE-only, and IKE-plus-PDT conditions. Metabolite signals were normalized to the corresponding untreated control for each cell line. Solid bars represent dark conditions, whereas checkered bars represent samples exposed to 850 nm NIR irradiation. Bars show mean ± SD, with individual biological replicates displayed. PS, photosensitizer; IKE, imidazole ketone erastin; NIR, near-infrared irradiation.

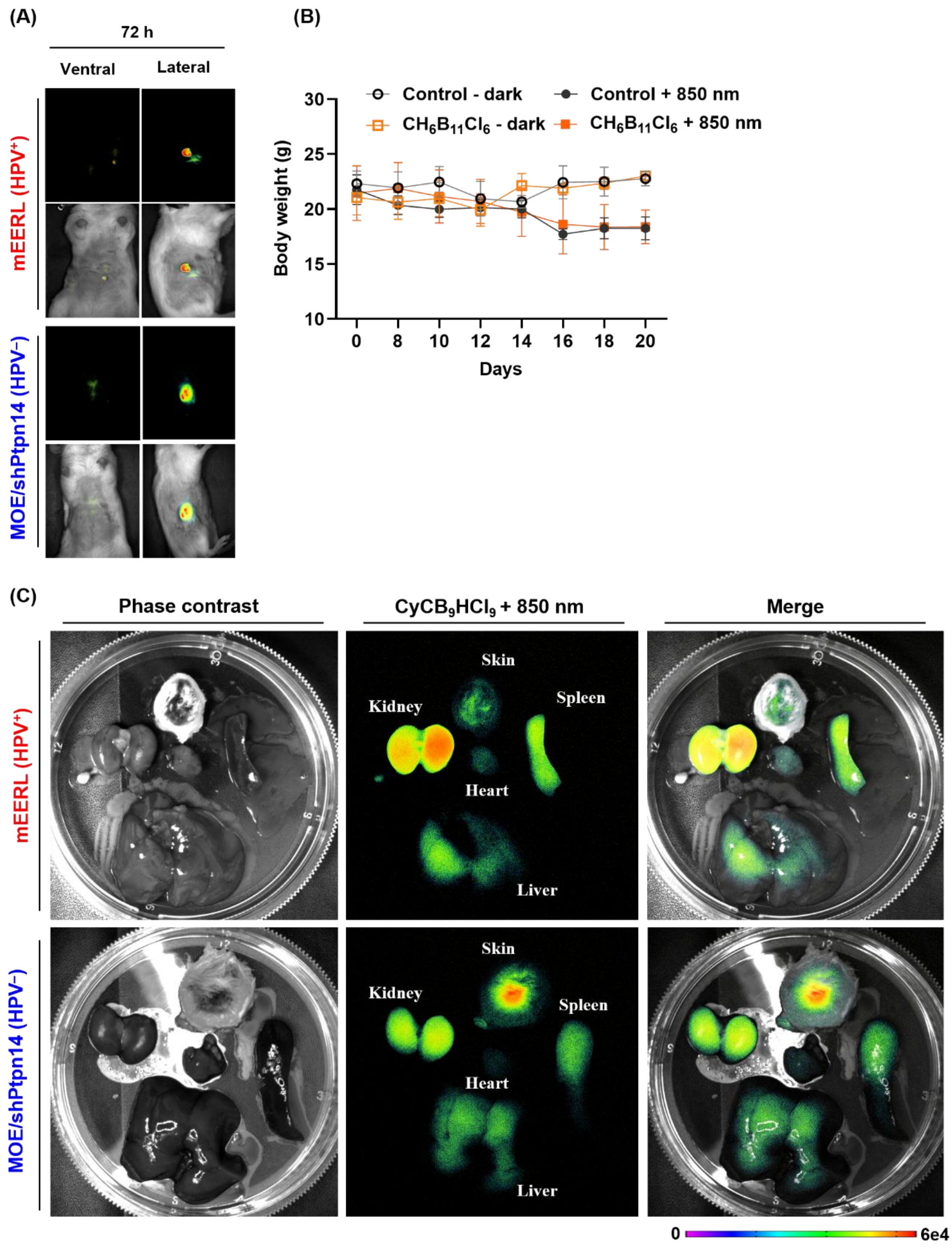

Figure S9. Extended *in vivo* photosensitizer retention and body weight monitoring following cyanine–carborate PDT. (A) Representative ventral and lateral fluorescence images of HPV-positive mEERL and HPV-negative MOE/shPtpn14 tumor-bearing mice 72 h after intravenous administration of $CyCB_{11}H_6Cl_6$. Fluorescence signal remained detectable at the flank tumor sites at 72 h post-injection. (B) Longitudinal body weight measurements of tumor-bearing mice treated with vehicle control under dark conditions, vehicle control with NIR irradiation, $CyCB_{11}H_6Cl_6$ under dark conditions, or $CyCB_{11}H_6Cl_6$ followed by NIR irradiation. Body weight remained stable across treatment groups, supporting limited overt systemic toxicity during the treatment period. Data are presented as mean ± S.D. (C) Representative phase

contrast, fluorescence, and merged images of kidneys, liver, spleen, heart, and excised skin/tumor-associated tissue collected at the experimental endpoint from mice bearing mEERL (HPV-positive; top row) or MOE/shPtpn14 (HPV-negative; bottom row) tumors. Fluorescence intensity is displayed using the pseudocolor scale shown.

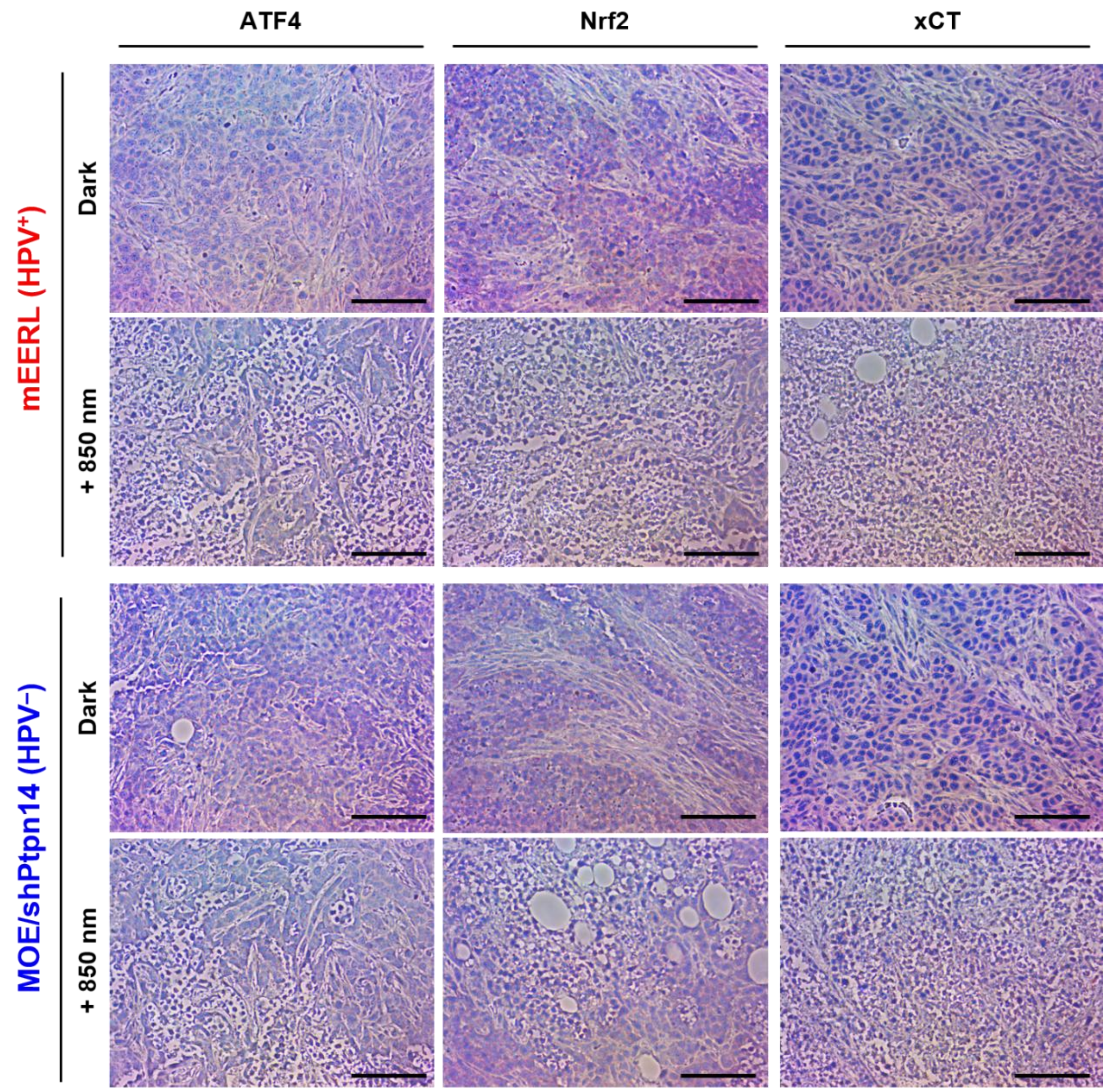

Figure S10. Immunohistochemical detection of ATF4, Nrf2, and xCT in HPV-positive and HPV-negative HNSCC tumors. Representative immunohistochemical images of mEERL (HPV-positive; top two rows) and MOE/shPtpn14 (HPV-negative; bottom two rows) tumor sections collected from dark-control and NIR-treated mice. AEC-positive immunoreactivity appears red-brown, and nuclei were counterstained blue with hematoxylin. Images are representative of tumors collected from n = 3 independent mice per treatment group. Scale bars: 400 µm.

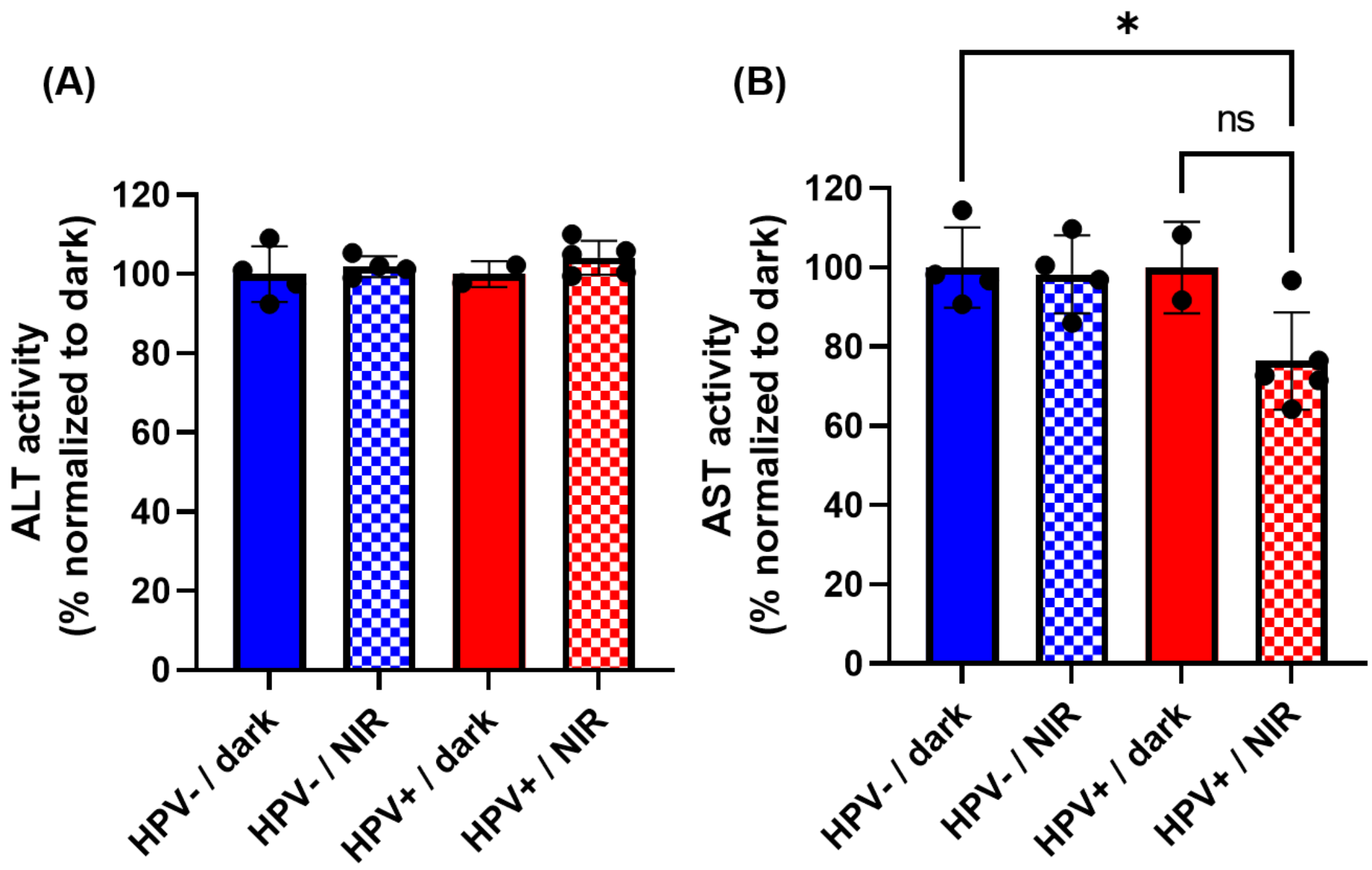

Figure S11. Serum ALT and AST activities following PDT in HPV-positive and HPV-negative HNSCC tumor-bearing mice. (A) Serum alanine aminotransferase (ALT) and (B) aspartate aminotransferase (AST) activities were measured in HPV-negative (HPV_{-}) and HPV-positive (HPV^{+}) tumor-bearing mice under dark or NIR irradiation conditions. Enzyme activities were normalized to the corresponding dark control and are presented as percentage of control. Data are presented as the mean ± S.D. (n = 4; *$P < 0.05$; ns, not significant).

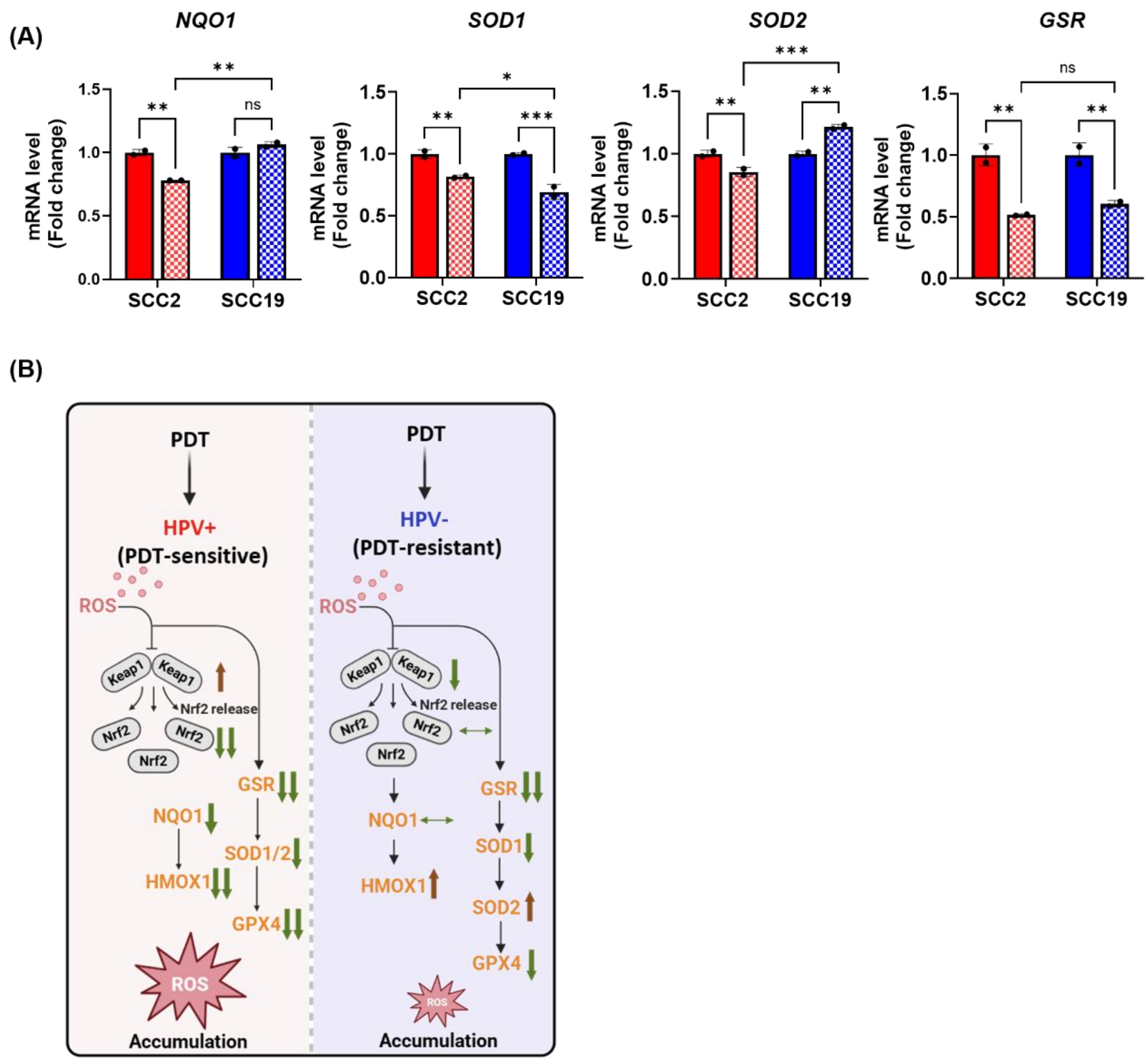

Figure S12. Differential antioxidant-response genes in PDT-sensitive and PDT-resistant HNSCC cells. (A) Quantitative PCR analysis of antioxidant- and redox-defense genes in PDT-sensitive SCC2 and PDT-resistant SCC19 cells under dark conditions or following NIR irradiation. (B) Working model summarizing differential antioxidant-response signaling following PDT. Upward arrows indicate increased expression, downward arrows indicate decreased expression, and horizontal/balanced arrows indicate little or no apparent change under the indicated condition. Data are presented as mean ± S.D.

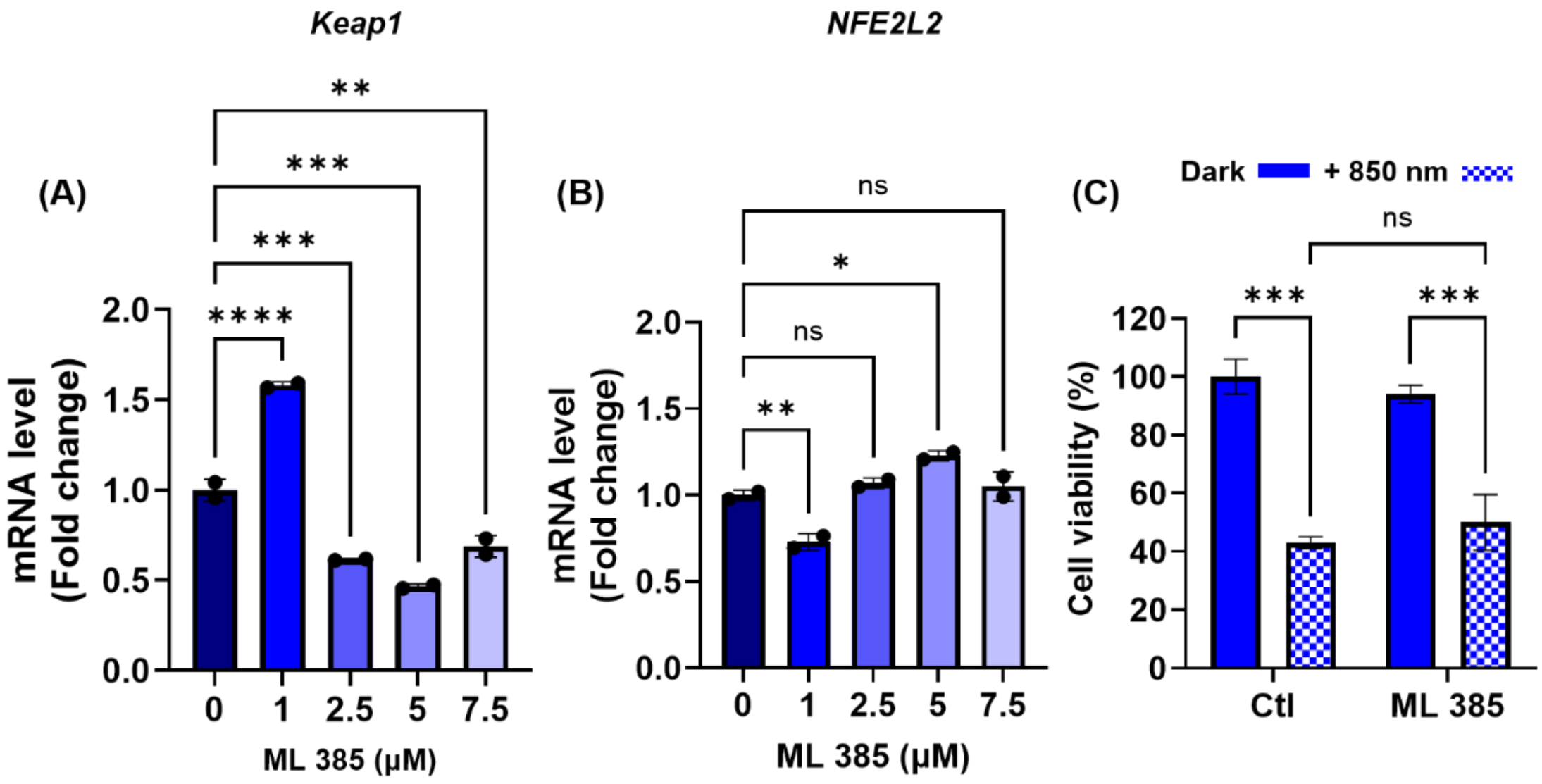

Figure S13. Nrf2 inhibition does not substantially sensitize PDT-resistant SCC19 cells to photodynamic stress. (A, B) Gene-expression analysis of Nrf2-associated redox-response markers in PDT-resistant SCC19 cells treated with the Nrf2 inhibitor (ML385). (C) Cell viability of SCC19 cells under dark conditions or following NIR irradiation in the presence or absence of ML385 after 48 h treatment. Data are presented as mean ± S.D.

Table S1. Sequences of primers used for real-time quantitative PCR.

Gene	Primer sequence
KEAP1	Sense: 5’-CCAAAGCGGGAATCATGTGC-3’<br>Antisense: 5’-CTGGAAGCCTGCTCTTTCCA-3’
NFE2L2	Sense: 5’-CAGGTTGCCCACATTCCCAA-3’<br>Antisense: 5’-CAAGTGACTGAAACGTAGCCG-3’
GPX4	Sense: 5’-CTCATCGACAAGAACGGCTG-3’<br>Antisense: 5’-GGAGGAAAGCTGGGTTAGGG-3’
HMOX-1	Sense: 5’-CCCCAACGAAAAGCACATCC-3’<br>Antisense: 5’-AGACAGCTGCCACATTAGGG-3’
RPLP0	Sense: 5’-TCCTCGTGGAAGTGACATCG-3’<br>Antisense: 5’-CTGTCTTCCCTGGGCATCAC-3’

Table S2. Half maximal inhibitory concentrations (IC_{50}) of cyanine-carborates with and without NIR irradiation in head and neck squamous cell carcinoma cell lines. IC_{50} values were generated by nonlinear regression analysis using GraphPad Prism. Error is displayed as a 95% confidence interval.

Cell lines	HPV status	NIR IC_{50} (μM)	Dark IC_{50} (μM)
SCC2	HPV^+	0.25 ± 0.14	0.97 ± 0.1
SCC90	HPV^+	0.47 ± 0.09	1.98 ± 0.23
SCC152	HPV^+	0.26 ± 0.11	1.57 ± 0.18
SCC1	HPV^-	0.49 ± 0.1	1.0 ± 0.2
SCC4	HPV^-	0.73 ± 0.12	1.1 ± 0.28
SCC19	HPV^-	0.86 ± 0.14	1.91 ± 0.11

Table S3. Quantitative histopathological scoring of tumor and major-organ necrosis following cyanine–carborate photodynamic therapy. H&E-stained sections of tumor, heart, kidney, liver, lung, and spleen collected from dark-control and NIR-treated mice were independently evaluated. Tissue necrosis was scored using the following scale: 0, no necrosis; 1, <25% necrosis; 2, 25–50% necrosis; 3, 51–75% necrosis; and 4, >75% necrosis. Individual scores and group means ± SD are presented for two sections per tissue and treatment group.

**Tissue**	**Dark-control scores**	**Dark control, mean ± SD**	**NIR-treated scores**	**NIR treated, mean ± SD**	**Microscopic observations**
Heart	0, 0	0.0 ± 0.0	0, 0	0.0 ± 0.0	No inflammation or other apparent histopathological abnormalities were observed in either group.
Kidney	0, 0	0.0 ± 0.0	0, 0	0.0 ± 0.0	Focal chronic inflammation was noted in one NIR-treated section but was not associated with a positive injury score.
Liver	0, 0	0.0 ± 0.0	0, 0	0.0 ± 0.0	Focal chronic inflammation was noted in one NIR-treated section but was not associated with a positive injury score.
Lung	0, 0	0.0 ± 0.0	0, 0	0.0 ± 0.0	Mild chronic inflammation was noted in both dark-control sections, whereas no inflammation was observed in the NIR-treated sections.
Spleen	0, 0	0.0 ± 0.0	0, 0	0.0 ± 0.0	No scored histopathological abnormalities were observed; inflammation assessment was not applicable.
Tumor	0, 0	0.0 ± 0.0	4, 4	4.0 ± 0.0	Pronounced treatment-associated disruption was observed in NIR-treated tumors.
